# Measurement Properties of Hand‐Held Dynamometry for Ankle Strength Assessment: A COSMIN‐Based Systematic Review and Meta‐Analysis

**DOI:** 10.1002/msc.70285

**Published:** 2026-10-24

**Authors:** Matheus Hissa Lourenço Ferreira, João Victor Völker Oliveira, Nelson Carvas Junior, Túlio Medina Dutra de Oliveira, Guilherme Augusto Santos Araujo, José Elias Filho, George Schayer Sabino, Carla Malaguti, Diogo Carvalho Felício

**Affiliations:** ^1^ Universidade Federal de Minas Gerais (UFMG) Belo Horizonte MG Brazil; ^2^ Universidade Federal de Juiz de Fora (UFJF) Juiz de Fora MG Brazil; ^3^ Universidade Paulista (UNIP) São Paulo SP Brazil; ^4^ Departamento de Pós‐Graduação em Saúde Baseada em Evidências Universidade Federal de São Paulo (UNIFESP) São Paulo SP Brazil; ^5^ Faculdade de Sete Lagoas (FACSETE) Sete Lagoas MG Brazil; ^6^ Faculdade Ciências Médicas de Minas Gerais (FCM‐MG) Belo Horizonte MG Brazil

**Keywords:** ankle, handheld dynamometry, muscle strength, reliability, validity

## Abstract

**Objective:**

This systematic review and meta‐analysis evaluated the measurement properties of handheld dynamometry (HHD) for assessing isometric ankle muscle strength in healthy individuals.

**Methods:**

A comprehensive search was conducted in PubMed, EMBASE, CINAHL, Web of Science, SPORTDiscus, PsycINFO, Scopus, and the Virtual Health Library, with no language or date restrictions. Observational and methodological studies assessing dorsiflexors, plantar flexors, invertors, or evertors using HHD were eligible if they reported reliability, measurement error, or construct validity against isokinetic dynamometry. Random‐effects meta‐analyses were performed by measurement property and testing configuration, following COSMIN guidance.

**Results:**

Twenty‐four studies were included. Inter‐rater reliability was good for dorsiflexors (ICC = 0.89, 95% CI 0.80–0.94), plantar flexors (ICC = 0.86, 95% CI 0.72–0.93), invertors (ICC = 0.79, 95% CI 0.66–0.87), and evertors (ICC = 0.75, 95% CI 0.46–0.90). Intra‐rater reliability ranged from good to excellent (ICC = 0.88–0.91). External fixation was associated with numerically higher inter‐rater ICCs for dorsiflexors and plantar flexors although subgroup differences were not statistically significant. Construct validity was strong for dorsiflexors (*r* = 0.87, 95% CI 0.42–0.98) but uncertain for plantar flexors (*r* = 0.72, 95% CI −0.20–0.96). Measurement error remained indeterminate due to the absence of an established minimal important change. Certainty of evidence was high for reliability and low for construct validity; measurement error was not graded.

**Conclusion:**

HHD provides reliable assessment of isometric ankle strength, including without external fixation. Evidence for construct validity, particularly for plantar flexors, and for measurement error remains limited.

## 1 Introduction

Lower limb strength is associated with balance control and improved functional capacity (Lord et al. 1995; Spink et al. 2010). Regarding the foot and ankle, reduced muscle strength may represent a predictive factor for falls (Menz et al. 2006; Mickle et al. 2009; Spink et al. 2010) and for an increased risk of sprains in athletes (Delahunt and Remus 2019; Fousekis et al. 2012). Accurate assessment of ankle muscle strength can provide valuable insights in both sports and clinical contexts, particularly for screening purposes, rehabilitation monitoring, injury prevention, and decision‐making regarding return to sport (Aguilaniu et al. 2024; Liu et al. 2022; Smith et al. 2021). The accuracy of these measurements enables the identification of specific functional deficits and the monitoring of intervention outcomes in complex musculoskeletal conditions (Kittelson et al. 2021; Rudisill et al. 2021).

Handheld dynamometers have gained prominence in clinical practice due to their accessibility, portability, and reduced cost (Keep et al. 2016). Despite their limitations in collecting more complex kinetic data, handheld dynamometers demonstrate good reliability in the assessment of various muscle groups, including ankle muscles (Stark et al. 2011), and facilitate evaluations in diverse settings such as clinics, sports centres, and home care environments (Chamorro et al. 2021).

However, an important gap remains in the literature regarding the standardisation of application protocols for these devices, particularly with respect to patient positioning, the use of external fixations, and reference criteria (Garcia and Souza 2020). Furthermore, although the reliability of measurements obtained with handheld dynamometers has been investigated in several contexts, construct validity has yielded heterogeneous results (Chamorro et al. 2017).

Therefore, a rigorous synthesis of the current evidence is warranted. This systematic review aims to evaluate the measurement properties of handheld dynamometry (HHD) for isometric ankle muscle strength (dorsiflexion/plantar flexion, inversion/eversion) in adults, including reliability, measurement error, and construct validity, as well as to synthesise the quality of evidence across testing configurations with and without fixation.

## 2 Methods

This study is a systematic review conducted in accordance with the Preferred Reporting Items for Systematic Reviews and Meta‐Analyses (PRISMA 2020) guidelines (Page et al. 2021) and based on the COSMIN methodological framework (Elsman et al. 2024). The study was registered in PROSPERO (CRD42042622199).

### 2.1 Search Strategy

Systematic searches were conducted in the PubMed, EMBASE, CINAHL, Web of Science, SPORTDiscus, PsycINFO, Scopus, and Virtual Health Library (BVS) databases, with no language restrictions. The initial search had no date restrictions. An updated search was performed on August 6, 2026, to identify studies published after the original search. The search strategy combined descriptors related to ankle isometric strength, handheld dynamometry, and measurement properties. Searches were adapted for each database. The original and updated search strategies are presented in Supporting Information S1: Tables S1 and S2, respectively. Duplicates were removed using the Rayyan platform (Ouzzani et al. 2016).

### 2.2 Eligibility Criteria

Study selection was guided by the PICOS strategy (Population, Intervention, Comparator, Outcome, and Study design). The population of interest comprised healthy individuals. Studies that included mixed samples of healthy and pathological participants were eligible only when data for the healthy subgroup could be extracted separately; studies that did not allow this separation were excluded. The intervention was defined as the measurement of isometric strength using handheld dynamometry of any brand, with or without external fixation, and employing either ‘make’ or ‘break’ protocols. Both testing approaches were included because they are commonly used in clinical practice; however, the inclusion of both protocols may have contributed to heterogeneity in the measurement properties observed across studies. When relevant, isokinetic dynamometry (IKD) was considered as the reference comparator within the scope of hypothesis testing, but only when the comparison involved isometric contractions performed under similar joint positions and angles. Studies that compared handheld dynamometry exclusively with concentric or eccentric isokinetic modes were excluded. Eligible outcomes included at least one measurement property: reliability (intraclass correlation coefficient, ICC), measurement error (standard error of measurement, SEM; minimal detectable change or smallest detectable change, MDC/SDC), construct validity (correlations or hypothesis testing), or responsiveness. The study design included observational or methodological studies reporting psychometric statistics, with no restrictions on language or date of publication. Studies that employed HHD solely as an outcome measure without reporting measurement properties were excluded.

### 2.3 Inclusion Criteria

Studies were included if they assessed the isometric strength of ankle muscles—dorsiflexors, plantar flexors, evertors, and invertors—using portable devices (HHD, portable load cells, dynamometers with fixation, or equivalent portable instruments) and reported at least one of the following measurement properties: intra‐ or inter‐rater reliability, measurement error (SEM, MDC/SDC), or construct validity (correlations or hypothesis testing), provided the comparator was isometric isokinetic dynamometry. Studies reporting only responsiveness were eligible in principle, but none provided sufficient data for inclusion in the synthesis.

### 2.4 Exclusion Criteria

Studies were excluded if they assessed participants with musculoskeletal, neurological, cardiovascular, or systemic pathological conditions that could compromise strength stability or test performance (unless data for healthy participants were reported separately); studies of eccentric/concentric contractions without emphasis on isometry; studies comparing HHD solely with concentric or eccentric isokinetic measurements; validations using non‐isokinetic comparators; clinical trials without psychometric data; reviews, dissertations, theses, or grey literature without sufficient data.

### 2.5 Data Extraction

Two reviewers (M.H.L.F. and J.V.V.) independently extracted the following data: author/year, sample and characteristics, type of fixation (external vs. absent), test type (make/break), muscle group, device, and psychometric estimates (ICC with model/measurement and 95% CI; SEM, MDC/SDC; HHD–IKD correlations). A third reviewer resolved potential disagreements (D.C.F.).

To minimise selection bias when multiple estimates were reported within a study, the following hierarchy was applied: (1) ICC model appropriate to the context (inter‐rater: ICC(2,1), absolute agreement; intra‐rater: ICC(3,1)); (2) single‐measure, unless the intended use was the mean of repeated trials; (3) highest‐quality protocol (standardized fixation, clearly defined position/angle, standardized contraction duration, and declared make/break test).

When a study reported results with and without fixation, these were treated as distinct arms by configuration to avoid double‐counting within the same meta‐analysis. In studies including multiple HHDs or several raters/days, priority was given to the arm that best met criteria 1–3; if dependency persisted, we used a multilevel model or selected a single estimate according to the hierarchy.

When only SEM was reported, MDC95 was estimated as SEM × 2.77; when only MDC was reported, SEM was estimated as MDC/2.77 (Wyrwich et al. 1999). For synthesis, original values and complete confidence intervals were used and rounding was applied only for results presentation. For laterality, preference was given to one limb per participant, prioritising the dominant limb when available, in order to avoid pseudo‐replication; in its absence, data were aggregated per participant whenever possible.

### 2.6 Methodological Quality Assessment (COSMIN Risk of Bias)

Methodological quality was assessed using the COSMIN tool (Mokkink et al. 2010), applying the relevant boxes for performance‐based instruments: reliability, measurement error, construct validity, cross‐cultural validity/invariance, when applicable, and responsiveness. Isokinetic dynamometry performed in isometric mode under comparable testing conditions was considered a reference method for comparison, without assuming that it constituted a definitive gold standard for isometric ankle strength. Accordingly, these comparisons were evaluated within the COSMIN framework of hypothesis testing for construct validity, rather than as criterion validity (Mokkink et al. 2010; Chamorro et al. 2017). Each study–property pair was rated as very good, adequate, doubtful, or inadequate, following the worst‐score‐count rule. Two reviewers (M.H.L.F. and J.V.V.) independently conducted the assessment, and disagreements were resolved by a third reviewer (D.C.F.) (Mokkink et al. 2010).

### 2.7 Definition of Properties and Sufficiency Criteria (COSMIN)

The sufficiency criteria were pre‐specified. Reliability was considered sufficient (+) when ICC ≥ 0.70 for group‐level use or ≥ 0.90 for individual decision‐making. Measurement error (SEM, MDC95) was deemed sufficient when MDC ≤ minimal important change (MIC) or SDC ≤ MIC; in the absence of an MIC, the judgement was rated as indeterminate (?). Construct validity was considered sufficient when ≥ 75% of a priori hypotheses were confirmed: (H1) *r* (HHD vs. IKD) ≥ 0.70 for dorsiflexion; (H2) stronger correlations with external fixation compared to no fixation; (H3) higher similarity of protocols between methods resulting in higher correlations; (H4) correlations for plantar flexion tend to be lower than those for dorsiflexion (Mokkink et al. 2024).

### 2.8 Quality of Evidence (GRADE‐COSMIN)

The certainty of the evidence was assessed using the GRADE approach adapted by COSMIN, by measurement property and testing configuration (e.g., with vs. without fixation), considering risk of bias, inconsistency, imprecision, and indirectness. When *k* ≥ 10, publication bias was explored. The final grading (high ⨁⨁⨁⨁, moderate ⨁⨁⨁◯, low ⨁⨁◯◯, very low ⨁◯◯◯) was accompanied by footnotes justifying any downgrading. Two reviewers (M.H.L.F. and J.V.V.) independently applied the GRADE assessment, and disagreements were resolved by a third reviewer (D.C.F.) (Prinsen et al. 2018).

### 2.9 Data Synthesis and Statistical Analysis

Meta‐analyses were conducted only when ≥ 2 studies provided comparable estimates for the same configuration, considering the ICC model, whether a single or average measure was used, the presence or absence of fixation, and the joint position or angle. When *k* < 2 per movement (e.g., inversion/eversion) or when comparability was not met, a narrative synthesis was adopted and ‘not assessed’ (NA) was indicated in the COSMIN figures.

The synthesis was performed separately for each measurement property and configuration. Intra‐ and inter‐rater reliability studies were analysed independently. ICC models were preserved and, when possible, stratified (e.g., ICC(2,1) for inter‐rater absolute agreement and ICC(3,1) for intra‐rater mixed‐effects) with respect to single‐measure versus average‐measure reporting. Each movement direction was analysed as a separate outcome, including dorsiflexion, plantar flexion, inversion and eversion.

For construct validity of HHD–IKD, Pearson's (r) or Spearman's (ρ) correlations were extracted according to data distribution. In meta‐analyses, r/ρ values were transformed using Fisher's z, pooled under random‐effects models, and back‐transformed to the original scale. ICCs were pooled under random‐effects models using ICC‐specific variance/standard error (SE), without Fisher's z transformation. When a study contributed multiple estimates for the same contrast, the independence rules described above were followed, either by selecting a single estimate per protocol or by applying multilevel models.

Heterogeneity was assessed with Cochran's Q test (*p* < 0.10 as indicative) and quantified using I^2^ and τ^2^ under random‐effects models (τ^2^ estimated by restricted maximum likelihood). In the presence of substantial heterogeneity, pre‐specified subgroup analyses were performed according to the presence or absence of external fixation. Although additional subgroup analyses (make vs. break protocols) and sensitivity analyses (leave‐one‐out, exclusion of studies with high risk of bias, and restriction to single‐measure estimates) had been planned, they were not performed due to the limited number of studies available for most individual contrasts and the high clinical heterogeneity across testing protocols. Prediction intervals and formal assessment of small‐study effects were likewise not calculated. Magnitude was interpreted according to Koo and Li (2016) for ICC (< 0.50 poor; 0.50–0.75 moderate; 0.75–0.90 good; > 0.90 excellent) and Mukaka (2012) for correlations, always considering 95% CIs and the clinical context.

When studies did not report the 95% confidence interval or standard error of the ICC, the variance was approximated using the formula based on the reported ICC value and sample size (Bonett 2002; Shrout and Fleiss 1979). Sensitivity analyses restricted to studies that reported complete confidence intervals were planned but not performed.

Analyses were performed in R version 4.5.1. Although sensitivity analyses (leave‐one‐out, exclusion of studies with high COSMIN risk of bias, and restriction to single‐measure estimates) and formal assessment of small‐study effects had been planned, they were not performed due to the limited number of studies available for most individual contrasts and the high clinical heterogeneity across testing protocols.

## 3 Results

The original search identified 5342 records. After removal of 978 duplicates, 4364 records were screened by title and abstract, and 4326 were excluded. Full‐text assessment was performed for 38 records (one could not be retrieved), leading to the exclusion of 17 studies. One additional study was identified through reference list screening. Twenty‐one studies met the eligibility criteria in the original search.

An updated search performed in August 2026 retrieved 968 new records (CINAHL = 342, Web of Science = 61, Scopus = 179, BVS = 141, SPORTDiscus = 114, PubMed = 77, Embase = 54). After removal of 152 duplicates, the remaining records were screened by title and abstract. Seven articles were assessed in full text: four were excluded and three met the inclusion criteria.

In total, 24 studies were included in the review (Figure 1). Studies excluded after full‐text assessment, with reasons, are presented in Supporting Information S1: Table S3.

**FIGURE 1:**
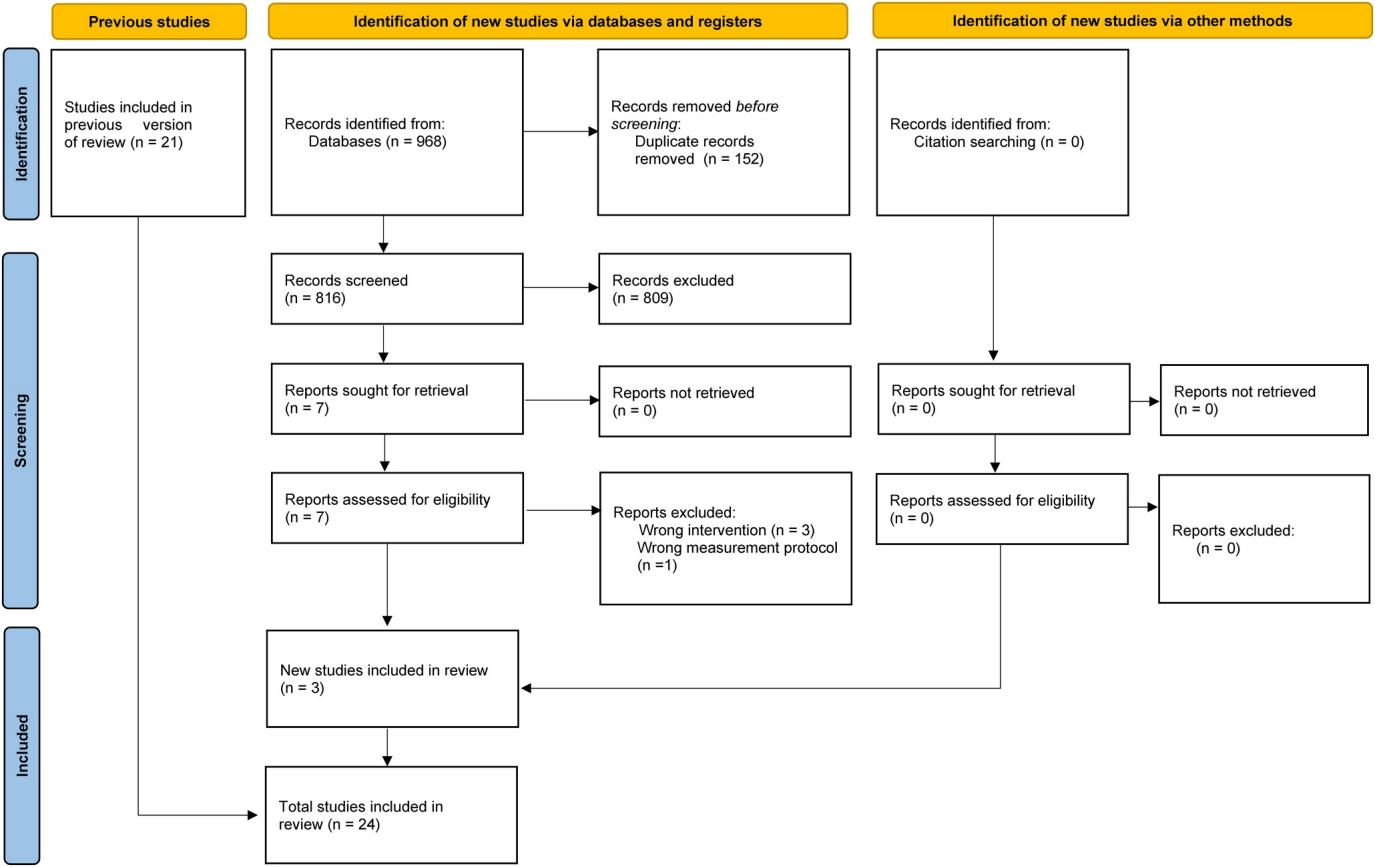
PRISMA flow diagram of study selection (original search + update in August 2026).

The characteristics and results of studies assessing intra‐rater and inter‐rater reliability of handheld dynamometry for isometric ankle strength are presented in Tables 1 and 2, respectively. The characteristics and results of studies evaluating construct validity between handheld dynamometry and isokinetic dynamometry are presented in Table 3.

**TABLE 1:** Intra‐rater reliability (ICC 3.1).

Author (year)	Sample (*n*)	Muscle group assessed	ICC	95% CI	SEM	MDC	HHD fixation/stabilisation
Buckinx et al. (2015)	30	Dorsiflexors	0.65	0.45–0.86	21.1 N	19.5 N	No external fixation
		Plantar flexors	0.60	0.34–0.83	36.4 N	29.6 N	No external fixation
Clarke et al. (2011)	38	Plantar flexors	0.88	0.78–0.94	18.9 N	52.3 N	Strap fixation placed just above the ankle
Daloia et al. (2018)	110	Dorsiflexors	0.95	—	—	—	No external fixation
		Plantar flexors	0.95	—	—	—	No external fixation
Ford‐Smith et al. (2001)	25	Dorsiflexors	0.71	0.50–0.84	—	—	Fixation using a metallic structure with locks
		Plantar flexors	0.84	0.71–0.92	—	—	Fixation using a metallic structure with locks
Jackson et al. (2017)	30	Plantar flexors	0.98	0.93–0.99	9.5 N	26.4 N	StabD (stabilisation device)
Kim and Lee (2015)	55	Dorsiflexors	0.97	0.91–0.97	—	—	No external fixation
		Plantar flexors	0.96	0.94–0.98	—	—	No external fixation
Romero‐Franco et al. (2017)	11	Dorsiflexors	0.97	0.89–0.99	70.6 N	195.4 N	Stabilised with straps and belts
		Plantar flexors	0.88	0.63–0.96	304.1 N	109.7 N	Stabilised with straps and belts
Schaeffer et al. (2021)	26	Dorsiflexors	0.89	0.78–0.95	1.0 N	3.5 N	No external fixation
		Invertors	0.88	0.76–0.95	1.0 N	1.8 N	No external fixation
		Evertors	0.89	0.77–0.95	1.0 N	1.8 N	No external fixation
Spink et al. (2010)	72	Dorsiflexors	0.91	0.86–0.94	14.1 N	39.0 N	No external fixation
		Plantar flexors	0.89	0.83–0.93	18.7 N	52.0 N	No external fixation
		Invertors	0.90	0.85–0.94	13.9 N	58.5 N	No external fixation
		Evertors	0.88	0.82–0.92	15.2 N	42.1 N	No external fixation
McGirr et al. (2014)	25	Invertors	0.80	0.59–0.90	—	—	No external fixation
		Evertors	0.86	0.71–0.94	—	—	No external fixation
Rawdon et al. (2025)	12	Plantar flexors	0.91	—	—	—	Anchored
		Dorsiflexors	0.76	—	—	—	Anchored
Espino et al. (2025)	10	Dorsiflexors	0.97	—	—	—	Belt‐stabilised
		Plantar flexors	0.99	—	—	—	Belt‐stabilised

Abbreviations: —, not reported; CI, confidence interval; HHD, hand‐held dynamometer; ICC, intraclass correlation coefficient; MDC, minimal detectable change; SEM, standard error of measurement.

**TABLE 2:** Inter‐rater reliability (ICC 2,1).

Author (year)	Sample (*n*)	Muscle group assessed	ICC	95% CI	SEM	MDC	HHD fixation/stabilisation
Buckinx et al. (2015)	30	Dorsiflexors	0.75	0.60–0.91	16.77 N	14.07 N	No external fixation
		Plantar flexors	0.62	0.41–0.84	37.99 N	24.42 N	No external fixation
Clarke et al. (2011)	38	Plantar flexors	0.23	0.03–0.45	—	—	Strap fixation placed just above the ankle
Daloia et al. (2018)	110	Dorsiflexors	0.94	—	—	—	No external fixation
		Plantar flexors	0.88	—	—	—	No external fixation
Hébert et al. (2011)	10	Dorsiflexors	0.67	0.11–0.91	—	—	Strap fixation
		Plantar flexors	0.87	0.56–0.97	—	—	Strap fixation
Katoh and Yamasaki (2009)	42	Dorsiflexors	0.98	0.96–0.99	—	—	Belt fixation
	44	Plantar flexors	0.99	0.98–0.99	—	—	Belt fixation
Kelln et al. (2008)	30	Dorsiflexors	0.86	—	0.45	1.25	Belt fixation
		Invertors	0.74	—	0.62	1.72	Belt fixation
		Evertors	0.84	—	0.52	1.44	Belt fixation
Kim and Lee (2015)	55	Dorsiflexors	0.90	0.84–0.94	—	—	No external fixation
		Plantar flexors	0.86	0.77–0.92	—	—	No external fixation
I. F. Kimura et al. (1996)	12	Dorsiflexors	0.43	—	—	—	No external fixation
M. Kimura et al. (2018)	30	Dorsiflexors	0.94	0.87–0.97	—	—	Belt fixation
Mentiplay et al. (2015)	30	Dorsiflexors	0.87	0.72–0.94	11.63%	32.24%	No external fixation
		Plantar flexors	0.89	0.76–0.95	10.64%	29.48%	No external fixation
McMahon et al. (1992)	20	Dorsiflexors	0.86	—	—	—	No external fixation
Romero‐Franco et al. (2017)	11	Dorsiflexors	0.99	0.97–0.99	38.2 N	105.8 N	Fixation with straps and belts
		Plantar flexors	0.87	0.51–0.98	116.6 N	323.0 N	Fixation with straps and belts
Sanada et al. (2008)	20	Plantar flexors	0.80	—	—	—	Belt fixation
Schaeffer et al. (2021)	26	Dorsiflexors	0.74	0.41–0.90	1.13 N	3.13 N	No external fixation
		Invertors	0.84	0.63–0.94	0.28 N	0.78 N	No external fixation
		Evertors	0.62	0.18–0.84	0.46 N	1.27 N	No external fixation
Shimizu et al. (2023)	20	Plantar flexors	0.94	0.86–0.97	65.37 N	181.1 N	Fixation with rope or straps
Nakagawa et al. (2025)	35	Plantar flexors	0.72	0.52–0.84	—	—	Belt and metal plate fixation
Espino et al. (2025)	10	Dorsiflexors	0.92	—	—	—	Belt‐stabilised
		Plantar flexors	0.98	—	—	—	Belt‐stabilised

*Note:* SEM and MDC values are reported as published by the original authors. In some studies (Buckinx et al. 2015; Romero‐Franco et al. 2017), the reported MDC is smaller than the SEM, indicating that a non‐standard formula or different confidence level may have been used.

Abbreviations: —, not reported; CI, confidence interval; HHD, hand‐held dynamometer; ICC, intraclass correlation coefficient; MDC, minimal detectable change; SEM, standard error of measurement.

**TABLE 3:** Construct validity (HHD–IKD correlation).

Author (year)	Sample (*n*)	Muscle group assessed	Pearson's r	95% CI	Reference instrument	HHD fixation/stabilisation
Mahaffey et al. (2022)	61	Dorsiflexors	0.51	0.29–0.67	Cybex II	Strap fixation
		Plantar flexors	0.15	−0.11–0.39	Cybex II	Strap fixation
Mauracher et al. (2019)	20	Dorsiflexors	0.90	—	IKD	External fixation with Velcro strap
Mentiplay et al. (2015)	30	Dorsiflexors	0.66	0.36–0.84	KinCom dynamometer	No external fixation
		Plantar flexors	0.51	0.14–0.75	KinCom dynamometer	No external fixation
Romero‐Franco et al. (2017)	11	Dorsiflexors	0.99	0.98–1.00	BIODEX system pro 4	Unspecified external fixation
		Plantar flexors	0.99	0.98–0.99	BIODEX system pro 4	Unspecified external fixation
Nakagawa et al. (2025)	22	Plantar flexors	0.34	−0.22–0.58	BIODEX system 3	Belt and metal plate fixation

Abbreviations: —, not reported; CI, confidence interval; HHD, hand‐held dynamometer; IKD, isokinetic dynamometer.

### 3.1 Meta‐Analysis

#### 3.1.1 Dorsiflexors ICC 2,1 Inter‐Rater

The meta‐analysis of studies investigating the inter‐rater reliability of dorsiflexor muscle strength included 13 studies (Buckinx et al. 2015; Daloia et al. 2018; Espino et al. 2025; Hébert et al. 2011; Katoh and Yamasaki 2009; Kelln et al. 2008; Kim and Lee 2015; I. F. Kimura et al. 1996; M. Kimura et al. 2018; McMahon et al. 1992; Mentiplay et al. 2015; Romero‐Franco et al. 2017; Schaeffer et al. 2021), with a total of 416 observations. The pooled ICC was 0.89 (95% CI: 0.80 to 0.94; *k* = 13), indicating good reliability. Overall heterogeneity was classified as high (I^2^ = 85.4%, *Q* = 82.4; *p* < 0.0001) (Figure 2A).

**FIGURE 2:**
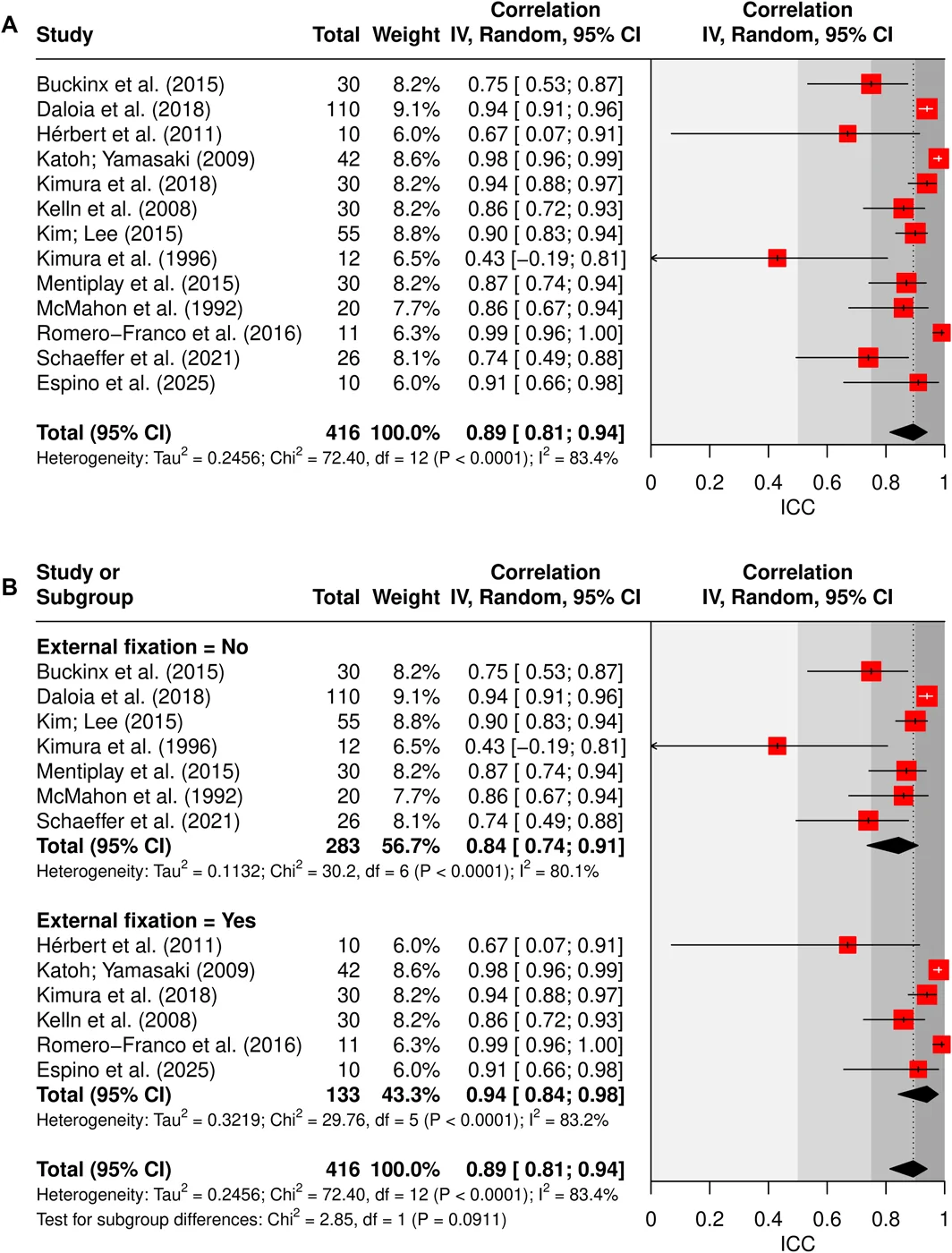
Forest plots summarising the inter‐rater reliability (ICC 2,1) of dorsiflexor muscle strength. Panel A presents the overall meta‐analysis including all studies. Panel B shows the subgroup analysis according to the presence or absence of external fixation.

#### 3.1.2 Dorsiflexors ICC 2,1 Inter‐Rater Subgroup Analysis According to the Presence of External Fixation

In the subgroup analysis based on the presence of external fixation, studies that employed some type of fixation demonstrated higher inter‐rater reliability (Espino et al. 2025; Hébert et al. 2011; Katoh and Yamasaki 2009; Kelln et al. 2008; M. Kimura et al. 2018; Romero‐Franco et al. 2017), with a pooled ICC of 0.94 (95% CI: 0.84 to 0.98, *k* = 6), indicating excellent reliability. In contrast, studies without fixation (Buckinx et al. 2015; Daloia et al. 2018; Kim and Lee 2015; I. F. Kimura et al. 1996; McMahon et al. 1992; Mentiplay et al. 2015; Schaeffer et al. 2021) showed a lower mean ICC of 0.84 (95% CI: 0.74 to 0.91, *k* = 7), representing good reliability. Heterogeneity was high in both subgroups (I^2^ = 80.4% and 85.1%, respectively). The difference between subgroups did not reach statistical significance (*Q* = 2.85; *p* = 0.09) (Figure 2B).

#### 3.1.3 Plantar Flexors ICC 2,1 Inter‐Rater

The meta‐analysis of studies assessing the inter‐rater reliability of plantar flexor muscle strength included 11 studies (Buckinx et al. 2015; Clarke et al. 2011; Daloia et al. 2018; Hébert et al. 2011; Katoh and Yamasaki 2009; Kim and Lee 2015; Mentiplay et al. 2015; Nakagawa et al. 2025; Romero‐Franco et al. 2017; Sanada et al. 2008; Shimizu et al. 2023), totaling 403 observations. The pooled ICC was 0.86 (95% CI: 0.72 to 0.93; *k* = 11), indicating good reliability. Heterogeneity among studies was considered high (I^2^ = 92.5%, *Q* = 132.87; *p* < 0.0001) (Figure 3A).

**FIGURE 3:**
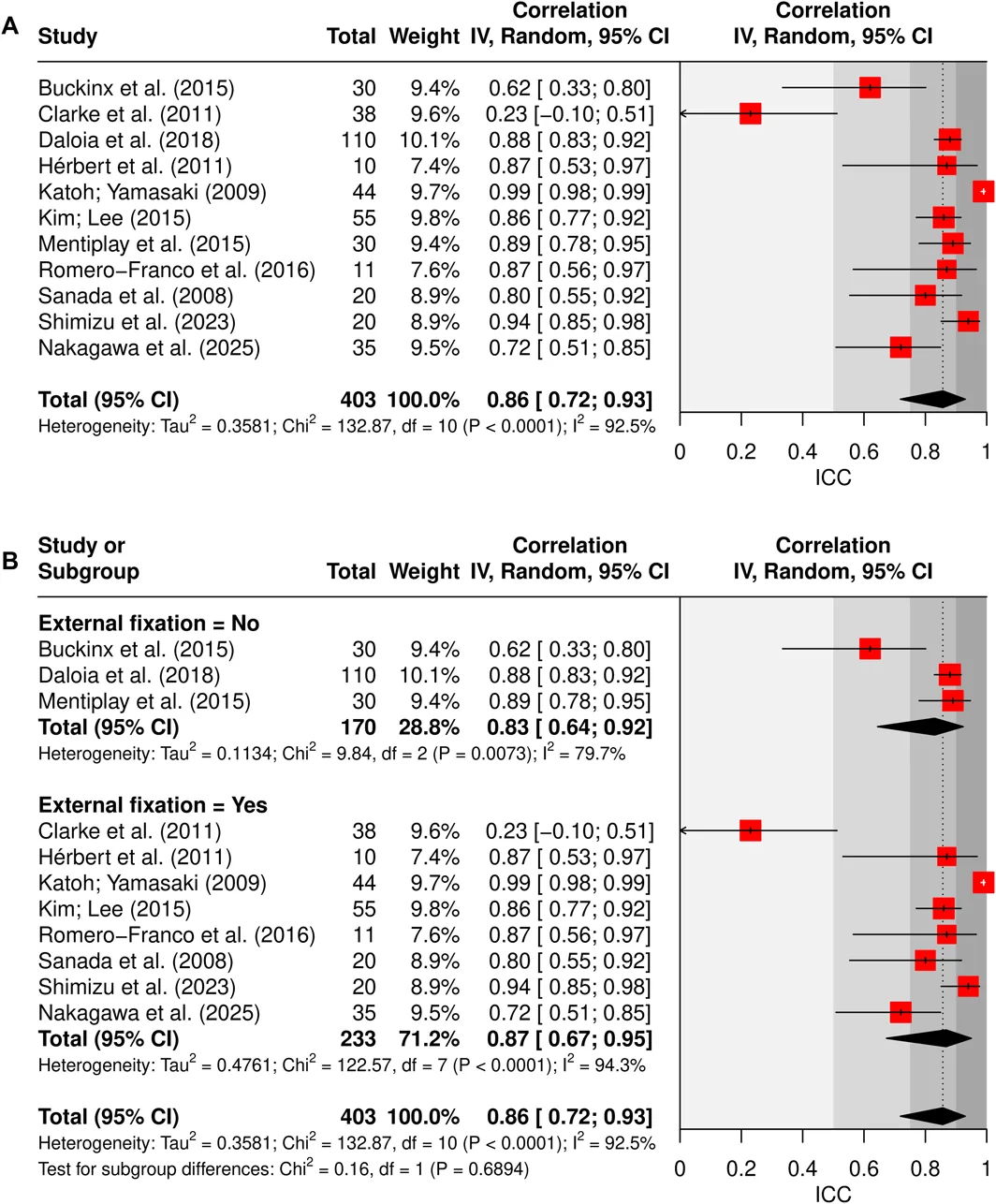
Forest plots summarising the inter‐rater reliability (ICC 2,1) of plantar flexor muscle strength. Panel A presents the overall meta‐analysis including all studies. Panel B shows the subgroup analysis according to the presence or absence of external fixation.

#### 3.1.4 Plantar Flexors ICC 2,1 Inter‐Rater Subgroup Analysis According to the Presence of External Fixation

Based on the subgroup analysis, studies using external fixation (Clarke et al. 2011; Hébert et al. 2011; Katoh and Yamasaki 2009; Kim and Lee 2015; Nakagawa et al. 2025; Romero‐Franco et al. 2017; Sanada et al. 2008; Shimizu et al. 2023) presented a pooled ICC of 0.87 (95% CI: 0.67 to 0.95, *k* = 8), which represents good reliability. Conversely, studies without fixation (Buckinx et al. 2015; Daloia et al. 2018; Mentiplay et al. 2015) reported a mean ICC of 0.83 (95% CI: 0.64 to 0.92, *k* = 3), also consistent with good reliability. Heterogeneity was high in both subgroups (I^2^ = 79.7% without fixation and 94.3% with fixation). The subgroup difference test was not statistically significant (*Q* = 0.16; *p* = 0.69), suggesting that the presence of external fixation did not result in a statistically detectable difference in inter‐rater reliability between the groups (Figure 3B).

#### 3.1.5 Invertors ICC 2,1 Inter‐Rater

The meta‐analysis of studies assessing the inter‐rater reliability of ankle invertor muscle strength included two studies (Kelln et al. 2008; Schaeffer et al. 2021), totaling 56 observations. The pooled ICC was 0.79 (95% CI: 0.66 to 0.87; *k* = 2), representing good reliability. Heterogeneity was considered null (I^2^ = 0%, *Q* = 0.91; *p* = 0.34) (Figure S1A). These results should be interpreted with caution given the small number of available studies (*k* = 2).

#### 3.1.6 Evertors ICC 2,1 Inter‐Rater

The meta‐analysis of studies evaluating the inter‐rater reliability of ankle evertor muscle strength included two studies (Kelln et al. 2008; Schaeffer et al. 2021), with a total of 56 observations. The pooled ICC was 0.75 (95% CI: 0.46 to 0.90; *k* = 2), indicating good reliability, although the wide confidence interval and lower bound approaching the moderate range suggest some uncertainty. Heterogeneity was substantial (I^2^ = 67.3%, *Q* = 3.06; *p* = 0.08) (Figure S1B). These results should be interpreted with caution given the small number of available studies (*k* = 2).

#### 3.1.7 Dorsiflexors ICC 3.1 Intra‐Rater

The meta‐analysis of studies evaluating the intra‐rater reliability of dorsiflexor muscle strength included seven studies (Buckinx et al. 2015; Daloia et al. 2018; Ford‐Smith et al. 2001; Kim and Lee 2015; Rawdon et al. 2025; Schaeffer et al. 2021; Spink et al. 2010), totaling 330 observations. The pooled ICC was 0.88 (95% CI: 0.77 to 0.94; *k* = 7), indicating good reliability. Heterogeneity among studies was considered high (I^2^ = 88.6%, *Q* = 52.76; *p* < 0.0001) (Figure S2A).

#### 3.1.8 Dorsiflexors ICC 3.1 Intra‐Rater Subgroup According to the Presence of External Fixation

In the subgroup analysis, studies without external fixation (Buckinx et al. 2015; Daloia et al. 2018; Kim and Lee 2015; Schaeffer et al. 2021; Spink et al. 2010) demonstrated a pooled ICC of 0.91 (95% CI: 0.81 to 0.96; *k* = 5), indicating excellent reliability. Studies using external fixation (Ford‐Smith et al. 2001; Rawdon et al. 2025) presented a pooled ICC of 0.72 (95% CI: 0.50 to 0.85; *k* = 2), representing moderate‐to‐good reliability. Heterogeneity was high in the no‐fixation subgroup (I^2^ = 89%) and absent in the fixation subgroup (I^2^ = 0%). The test for subgroup differences was statistically significant (*Q* = 5.13; *p* = 0.0235) (Figure S2B).

#### 3.1.9 Plantar Flexors ICC 3.1 Intra‐Rater

The meta‐analysis of studies evaluating the intra‐rater reliability of plantar flexor muscle strength included nine studies (Buckinx et al. 2015; Clarke et al. 2011; Daloia et al. 2018; Ford‐Smith et al. 2001; Jackson et al. 2017; Kim and Lee 2015; Rawdon et al. 2025; Romero‐Franco et al. 2017; Spink et al. 2010), totaling 383 observations. The pooled ICC was 0.91 (95% CI: 0.84 to 0.95; *k* = 9), indicating excellent reliability. Heterogeneity among studies was high (I^2^ = 85.4%, *Q* = 54.69; *p* < 0.0001) (Figure S3A).

#### 3.1.10 Plantar Flexors ICC 3.1 Intra‐Rater Subgroup According to the Presence of External Fixation

In the subgroup analysis, studies using external fixation (Clarke et al. 2011; Ford‐Smith et al. 2001; Jackson et al. 2017; Rawdon et al. 2025; Romero‐Franco et al. 2017) showed a pooled ICC of 0.92 (95% CI: 0.82 to 0.96; *k* = 5), classified as excellent. Studies without external fixation (Buckinx et al. 2015; Daloia et al. 2018; Kim and Lee 2015; Spink et al. 2010) also demonstrated excellent reliability, with a pooled ICC of 0.90 (95% CI: 0.74 to 0.97; *k* = 4). Heterogeneity was high in both subgroups (I^2^ = 91.7% without fixation and 78.3% with fixation). The test for subgroup differences was not statistically significant (*Q* = 0.08; *p* = 0.78) (Figure S3B).

#### 3.1.11 Ankle Invertors ICC 3,1 Intra‐Rater

Three studies (McGirr et al. 2014; Schaeffer et al. 2021; Spink et al. 2010), totaling 123 observations. The pooled ICC was 0.88 (95% CI: 0.72 to 0.95; *k* = 3), indicating good reliability. Heterogeneity was low (I^2^ = 14.1%, *Q* = 2.33; *p* = 0.31) (Figure S4A).

#### 3.1.12 Ankle Evertors ICC 3,1 Intra‐Rater

The meta‐analysis of studies evaluating the intra‐rater reliability of ankle evertor muscle strength included three studies (McGirr et al. 2014; Schaeffer et al. 2021; Spink et al. 2010), totaling 123 observations. The pooled ICC was 0.88 (95% CI: 0.83 to 0.91; *k* = 3), indicating good to excellent reliability. Heterogeneity was null (I^2^ = 0%, *Q* = 0.19; *p* = 0.91) (Figure S4B).

#### 3.1.13 Dorsiflexors Construct Validity

The meta‐analysis of four studies (Mahaffey et al. 2022; Mauracher et al. 2019; Mentiplay et al. 2015; Romero‐Franco et al. 2017), totaling 122 observations, indicated a pooled correlation of 0.87 (95% CI: 0.42 to 0.98; *k* = 4) between handheld dynamometry and isokinetic dynamometry, representing a strong correlation. High heterogeneity was observed among studies (I^2^ = 91.9%, *Q* = 36.87; *p* < 0.0001). Individual study correlations ranged from moderate (*r* = 0.51) to very strong (*r* = 0.99) (Figure 4A).

**FIGURE 4:**
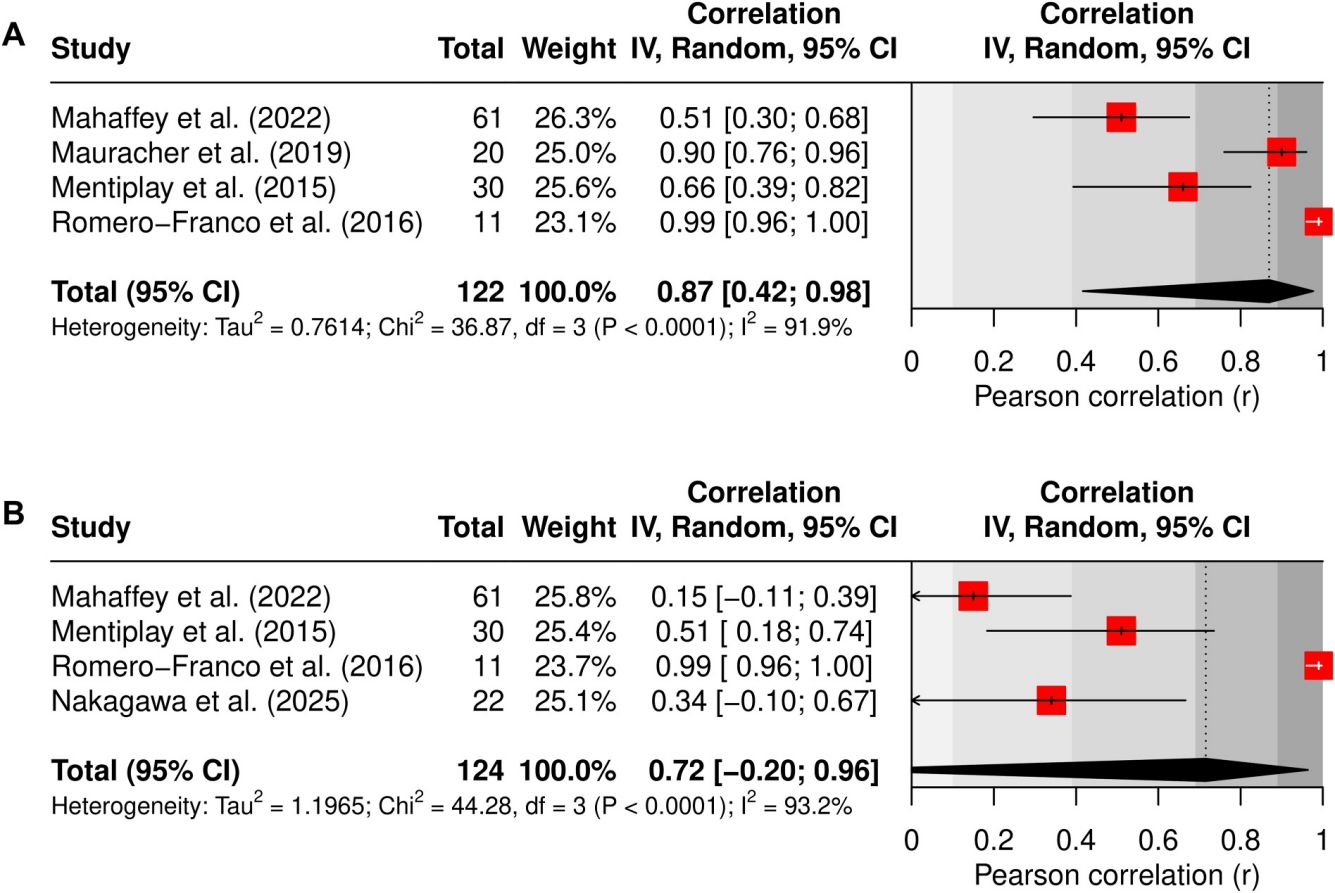
Forest plots summarising the construct validity of ankle dorsiflexor and plantar flexor muscle strength are shown in Panels A and B, respectively.

#### 3.1.14 Plantar Flexors Construct Validity

The meta‐analysis of four studies (Mahaffey et al. 2022; Mentiplay et al. 2015; Nakagawa et al. 2025; Romero‐Franco et al. 2017), totaling 124 observations, showed a pooled correlation of 0.72 (95% CI: −0.20 to 0.96; *k* = 4). The wide confidence interval crossing zero indicates that the correlation is uncertain and not statistically robust. Heterogeneity was very high (I^2^ = 93.2%, *Q* = 44.28; *p* < 0.0001) (Figure 4B).

#### 3.1.15 Reliability Pooled Summary

The classification of study reliability, based on ICC values, demonstrated consistent levels of precision. For dorsiflexor muscle strength, inter‐rater reliability was 0.89 (ICC [2, 1]; 95% CI: 0.80–0.94) and intra‐rater reliability was 0.88 (ICC [3, 1]; 95% CI: 0.77–0.94), both classified as good. Regarding plantar flexor muscles, inter‐rater reliability was 0.86 (ICC [2, 1]; 95% CI: 0.72–0.93), considered good, whereas intra‐rater reliability reached 0.91 (ICC [3, 1]; 95% CI: 0.84–0.95), classified as excellent.

For inversion and eversion movements, inter‐rater reliability was good for invertors (ICC = 0.79; 95% CI: 0.66–0.87) and evertors (ICC = 0.75; 95% CI: 0.46–0.90). Intra‐rater reliability (ICC [3, 1]) was good for both invertors (ICC = 0.88; 95% CI: 0.72–0.95) and evertors (ICC = 0.88; 95% CI: 0.83–0.91). Although the pooled values are high, the relatively small number of studies on invertors and evertors warrants cautious interpretation. Study‐level statistics are detailed in Supporting Information S1: Table S4.

#### 3.1.16 Construct Validity Pooled Summary

The construct validity analyses indicated a strong correlation for dorsiflexor strength (*r* = 0.87; 95% CI: 0.42–0.98) when compared with isokinetic dynamometry. For plantar flexors, the pooled correlation was *r* = 0.72 (95% CI: −0.20–0.96); however, the wide confidence interval, including negative values, reflects substantial uncertainty, preventing a definitive classification. Study‐level statistics are detailed in Supporting Information S1: Table S5.

### 3.2 Cosmin Risk of Bias

Based on the distribution of ratings, with 50.0% of studies classified as Adequate, 25.0% as Doubtful, 12.5% as Inadequate, and 12.5% as Very Good, the evaluated studies demonstrated an overall moderate methodological quality. Half of the studies were rated as Adequate, generally showing consistent procedures, including appropriate use of intraclass correlation coefficients, suitable testing intervals, and standardized protocols. However, these studies presented moderate limitations, mainly small sample sizes (< 50 participants) or a lack of assessor blinding. Only 12.5% achieved a Very Good rating, reflecting greater methodological rigour, adequate sample sizes and testing intervals, and a low risk of examiner‐related bias. Conversely, 25.0% were rated as Doubtful and 12.5% as Inadequate because of incomplete reporting, undefined test–retest intervals, small sample sizes, or statistical limitations (such as failure to report 95% confidence intervals, standard errors of measurement, or minimal detectable changes). Overall, reliability studies involving handheld dynamometry showed a low‐to‐moderate risk of bias, with relatively few meeting the highest methodological standards according to the COSMIN criteria (Supporting Information S1: Table S6).

### 3.3 Sufficiency Judgement

The sufficiency judgements of the measurement properties are summarised in Supporting Information S1: Table S7. Reliability was predominantly rated as sufficient, with 21 studies receiving a sufficient (+) rating and one receiving an insufficient (−) rating. For measurement error, the absence of an established minimal important change (MIC) precluded a definitive judgement of sufficiency; therefore, available measurement‐error results were considered indeterminate (?) according to COSMIN criteria. Construct validity showed mixed findings across studies and muscle groups, with stronger evidence for dorsiflexion than for plantar flexion.

### 3.4 Certainty of the Evidence

The certainty of evidence was evaluated using the GRADE approach adapted by COSMIN, considering the risk of bias, inconsistency, imprecision, and indirectness. Reliability was evaluated in 22 studies and showed predominantly sufficient results. Given the consistency of the overall sufficiency ratings, the adequate cumulative sample size, and the availability of multiple studies of at least adequate methodological quality, the certainty of evidence for reliability was rated as high.

Measurement error was reported in several studies using the standard error of measurement (SEM) and/or minimal detectable change (MDC). However, no established minimal important change (MIC) was available against which the measurement error could be compared. Therefore, according to the COSMIN criteria, the summarised measurement error was rated as indeterminate (?), and no GRADE certainty level was assigned.

Construct validity was evaluated using comparisons between hand‐held dynamometry and isokinetic dynamometry. Five unique studies contributed to these analyses. Although the pooled correlation was strong for dorsiflexion (*r* = 0.87, 95% CI 0.42–0.98), substantial between‐study heterogeneity was observed (I^2^ = 91.9%). For plantar flexion, the pooled correlation was *r* = 0.72 (95% CI −0.20–0.96), with very high heterogeneity (I^2^ = 93.2%) and a confidence interval crossing zero. Owing to the substantial and unexplained inconsistency across studies and muscle groups, the certainty of evidence for construct validity was downgraded by two levels and rated as low (Supporting Information S1: Table S8).

## 4 Discussion

This systematic review and meta‐analysis aimed to examine the reliability and construct validity of handheld dynamometry for measuring the isometric strength of ankle muscles. The results showed good to excellent intra‐ and inter‐rater reliability, especially for especially for dorsiflexors and plantar flexors. Construct validity showed strong correlations for dorsiflexors but uncertain evidence for plantar flexors.

Intra‐rater reliability for dorsiflexors (ICC: 0.88) and plantar flexors (ICC: 0.91) ranged from good to excellent. Although these values suggest that handheld dynamometry may be useful for monitoring muscle strength over time when assessed by the same examiner, the limited evidence on measurement error warrants a more cautious interpretation regarding its ability to detect meaningful individual changes. Inter‐rater reliability was also good for dorsiflexors and plantar flexors; however, the comparatively smaller body of evidence for invertors and evertors, together with the limited data on measurement error, indicates that interchangeable use across examiners should be interpreted with caution, particularly for these muscle groups.

These findings corroborate previous studies reporting high levels of reliability for handheld dynamometry in the lower limbs (Chamorro et al. 2021; Stark et al. 2011). Moreover, it was observed that the use of external fixation contributed to higher ICC values (for instance, for dorsiflexors in inter‐rater testing, ICC of 0.94 with fixation vs. 0.84 without fixation), as also reported by Waiteman et al. (2023), who investigated the reliability of dynamometry in the hip (Waiteman et al. 2023). Nevertheless, even without external fixation, reliability values remained good, which is clinically relevant, since in settings with time or resource constraints, handheld dynamometry without stabilisation may still provide valid measurements to promote the widespread use of objective muscle strength assessment.

Construct validity for dorsiflexors was classified as strong (*r*: 0.87), indicating good agreement between handheld dynamometry and isokinetic dynamometry, used as the comparator for hypothesis testing. However, no correlation was found for plantar flexors. The variability reflected in the wide 95% CI may be attributed to heterogeneity among the included studies, which involved differences in measurement protocols such as subject positioning, contraction duration, verbal instructions, inconsistencies in the use and type of external fixation, as well as divergences in the models of isokinetic dynamometers used as reference (e.g., Cybex, KinCom, Biodex). Therefore, handheld dynamometry appears to be a valid method for assessing dorsiflexor strength, whereas its equivalence for plantar flexors cannot yet be established based on the current evidence.

The findings regarding construct validity partially corroborate the existing literature. Mentiplay et al. (2015) found a strong correlation between handheld dynamometry and isokinetic dynamometry in the assessment of dorsiflexor peak force, consistent with the findings of the present study (Mentiplay et al. 2015). In contrast, the authors also reported a moderate‐to‐strong correlation for plantar flexors, which was not observed here. Marmon et al. (2013), in line with the current results, concluded that plantar flexor strength measures obtained with handheld dynamometry differ from those acquired with isokinetic dynamometry, that is, showing no correlation, and are likely influenced by the examiner's strength, since no external fixation was used (Marmon et al. 2013). According to Brinkmann (1994), when measuring high muscle forces with handheld dynamometers, the strength of the examiner may limit the results, as they must resist or overcome the force of the participant (Brinkmann 1994). The author also highlighted the challenges of stabilising both the dynamometer and the patient, particularly in the absence of external fixation, where the examiner serves as the only source of resistance (Brinkmann 1994). This may compromise the accuracy of measurements in stronger muscle groups, such as the plantar flexors. Similarly, Stark et al. (2011), in their systematic review, emphasised that the lack of standardisation in participant and examiner positioning, as well as in the applied force, undermines the validity of handheld dynamometry (Stark et al. 2011). The authors suggested that the implementation of standardized techniques is essential to mitigate these limitations and ensure greater reliability, especially in joints requiring higher stabilisation, as is the case for dorsiflexion compared with plantar flexion. On the other hand, although these methodological aspects are relevant, in the present study it was observed that intra‐rater reliability for plantar flexors was excellent even without external fixation (ICC = 0.90), being only slightly higher with fixation (ICC = 0.92). These findings suggest that although stabilisation may optimise standardisation, in clinical practice, the absence of fixation already provides reliable values.

Future research should prioritise the standardisation of handheld dynamometer assessment protocols, fixation methods, and examiner guidance. Expanding investigations to different populations, such as older adults, elite athletes, and individuals undergoing rehabilitation in either pre‐ or post‐injury phases, is also recommended to verify clinical applicability in specific contexts. In addition, there is a need for longitudinal studies exploring the responsiveness and predictive validity of the device.

The high heterogeneity among the studies, even after subgroup analysis, may be related to the lack of standardisation in measurement protocols, variations in the devices used, and differences in examiner training. The inclusion of both make and break testing protocols although reflective of clinical practice, may also have contributed to the high heterogeneity observed in some pooled estimates. Furthermore, the evidence on construct validity remains limited, particularly for movements such as inversion and eversion. It is important that the results presented here be interpreted with caution, especially regarding the applicability of the data to different clinical populations and in longitudinal assessments.

Although statistical heterogeneity was high for most reliability estimates, the direction of effects was consistent and the lower bounds of the confidence intervals for dorsiflexors and plantar flexors remained in the good range. Therefore, the certainty of evidence for reliability was not downgraded for inconsistency. In contrast, the very wide confidence intervals and inconsistent correlations for construct validity, particularly for plantar flexors, justified a two‐level downgrade.

The findings of this review support the feasibility of using handheld dynamometry for ankle strength assessment, particularly for dorsiflexors and plantar flexors, even in the absence of external fixation. Evidence for invertors and especially evertors remains more limited, and this should be taken into account when generalising the results to all ankle muscle groups. These results are particularly relevant in settings with structural limitations, such as home care or outpatient environments with restricted resources.

## 5 Conclusion

Handheld dynamometry demonstrated good to excellent reliability for assessing the isometric strength of ankle muscles, particularly for dorsiflexors and plantar flexors. Although the findings support its use in clinical and rehabilitation settings as a practical alternative to isokinetic dynamometers, the limited evidence on measurement error and the weaker body of evidence for invertors and evertors indicate that conclusions regarding the monitoring of individual changes over time and interchangeable use across examiners should be drawn with caution. Even without external fixation, reliability values remained acceptable for most muscle groups; however, further research is needed to strengthen the evidence for invertors and evertors and to better characterise measurement error.

## Author Contributions

J.V.V.O., G.S.S. and D.C.F. were responsible for the conception and design of the study. M.H.L.F., J.V.V.O. and J.E.F. were responsible for data collection. M.H.L.F. and N.C.J. were involved in the processing and statistical analysis of data. M.H.L.F., J.V.V.O., N.C.J., T.M.D.O., G.A.S.A., J.E.F., G.S.S., C.M. and D.C.F. were involved in the draughting of the manuscript, and all authors contributed to the interpretation of the data for the work and revising it critically for important intellectual content. M.H.L.F. takes responsibility for the integrity of the work. All authors have read and agreed to the published version of the manuscript.

## Funding

This work was funded by the Coordenação de Aperfeiçoamento de Pessoal de Nível Superior (CAPES)—Finance Code 001.

## Ethics Statement

This systematic review and meta‐analysis was based on data from previously published randomized controlled trials. No primary data were collected directly from human participants. Therefore, ethical approval was not required.

## Conflicts of Interest

The authors declare no conflicts of interest.

## Supporting information


Supporting Information S1

## Data Availability

The data supporting the findings of this study are available from the corresponding author upon request. Public access to these data is restricted for privacy or ethical reasons.
