# Supplementary material for: Measurement Properties of Hand‐Held Dynamometry for Ankle Strength Assessment: A COSMIN‐Based Systematic Review and Meta‐Analysis
Source: Musculoskeletal Care. 2026 Oct 24;24(4):e70285. doi: 10.1002/msc.70285 (PMC13695021; doi:10.1002/msc.70285)
Supplement: Supplementary file 1 [file MSC-24-e70285-s001.docx]

**Supplementary files**

**Supplementary Table S1.** Search strategy

| PubMed | (((((instrumentation[sh] OR methods[sh] OR "Validation Studies"[pt] OR "Comparative Study"[pt] OR "psychometrics"[MeSH] OR psychometr*[tiab] OR clinimetr*[tw] OR clinometr*[tw] OR "outcome assessment (health care)"[MeSH] OR "outcome assessment"[tiab] OR "outcome measure*"[tw] OR "observer variation"[MeSH] OR "observer variation"[tiab] OR "Health Status Indicators"[Mesh] OR "reproducibility of results"[MeSH] OR reproducib*[tiab] OR "discriminant analysis"[MeSH] OR reliab*[tiab] OR unreliab*[tiab] OR valid*[tiab] OR "coefficient of variation"[tiab] OR coefficient[tiab] OR homogeneity[tiab] OR homogeneous[tiab] OR "internal consistency"[tiab] OR (cronbach*[tiab] AND (alpha[tiab] OR alphas[tiab])) OR (item[tiab] AND (correlation*[tiab] OR selection*[tiab] OR reduction*[tiab])) OR agreement[tw] OR precision[tw] OR imprecision[tw] OR "precise values"[tw] OR test-retest[tiab] OR (test[tiab] AND retest[tiab]) OR (reliab*[tiab] AND (test[tiab] OR retest[tiab])) OR stability[tiab] OR interrater[tiab] OR inter-rater[tiab] OR intrarater[tiab] OR intra-rater[tiab] OR intertester[tiab] OR inter-tester[tiab] OR intratester[tiab] OR intra-tester[tiab] OR interobserver[tiab] OR inter-observer[tiab] OR intraobserver[tiab] OR intra-observer[tiab] OR intertechnician[tiab] OR inter-technician[tiab] OR intratechnician[tiab] OR intra-technician[tiab] OR interexaminer[tiab] OR inter-examiner[tiab] OR intraexaminer[tiab] OR intra-examiner[tiab] OR interassay[tiab] OR inter-assay[tiab] OR intraassay[tiab] OR intra-assay[tiab] OR interindividual[tiab] OR inter-individual[tiab] OR intraindividual[tiab] OR intra-individual[tiab] OR interparticipant[tiab] OR inter-participant[tiab] OR intraparticipant[tiab] OR intra-participant[tiab] OR kappa[tiab] OR kappa’s[tiab] OR kappas[tiab] OR repeatab*[tw] OR ((replicab*[tw] OR repeated[tw]) AND (measure[tw] OR measures[tw] OR findings[tw] OR result[tw] OR results[tw] OR test[tw] OR tests[tw])) OR generaliza*[tiab] OR generalisa*[tiab] OR concordance[tiab] OR (intraclass[tiab] AND correlation*[tiab]) OR discriminative[tiab] OR "known group"[tiab] OR "factor analysis"[tiab] OR "factor analyses"[tiab] OR "factor structure"[tiab] OR "factor structures"[tiab] OR dimension*[tiab] OR subscale*[tiab] OR (multitrait[tiab] AND scaling[tiab] AND (analysis[tiab] OR analyses[tiab])) OR "item discriminant"[tiab] OR "interscale correlation*"[tiab] OR error[tiab] OR errors[tiab] OR "individual variability"[tiab] OR "interval variability"[tiab] OR "rate variability"[tiab] OR (variability[tiab] AND (analysis[tiab] OR values[tiab])) OR (uncertainty[tiab] AND (measurement[tiab] OR measuring[tiab])) OR "standard error of measurement"[tiab] OR sensitiv*[tiab] OR responsive*[tiab] OR (limit[tiab] AND detection[tiab]) OR "minimal detectable concentration"[tiab] OR interpretab*[tiab] OR ((minimal[tiab] OR minimally[tiab] OR clinical[tiab] OR clinically[tiab]) AND (important[tiab] OR significant[tiab] OR detectable[tiab]) AND (change[tiab] OR difference[tiab])) OR (small*[tiab] AND (real[tiab] OR detectable[tiab]) AND (change[tiab] OR difference[tiab])) OR "meaningful change"[tiab] OR "ceiling effect"[tiab] OR "floor effect"[tiab] OR "Item response model"[tiab] OR IRT[tiab] OR Rasch[tiab] OR "Differential item functioning"[tiab] OR DIF[tiab] OR "computer adaptive testing"[tiab] OR "item bank"[tiab] OR "cross-cultural equivalence"[tiab])) AND (talocrural OR "ankle joint" OR foot OR ankle)) AND (handheld OR dynamometer OR isokinetic OR 'hand held' OR 'hand-held')) AND (flexion OR extension OR abduction OR (internal AND rotation) OR (rotation AND external) OR rotation OR adduction OR pronation OR supination OR eversion OR inversion OR (plantar flexion) OR dorsiflexion)) AND (strength OR muscle OR musculoskeletal OR isometric OR contraction) |
| --- | --- |
| EMBASE | #1 ('intermethod comparison'/exp OR 'data collection method'/exp OR 'validation study'/exp OR 'feasibility study'/exp OR 'pilot study'/exp OR 'psychometry'/exp OR 'reproducibility'/exp OR reproducib*:ab,ti OR 'audit':ab,ti OR psychometr*:ab,ti OR clinimetr*:ab,ti OR clinometr*:ab,ti OR 'observer variation'/exp OR 'observer variation':ab,ti OR 'discriminant analysis'/exp OR 'validity'/exp OR reliab*:ab,ti OR valid*:ab,ti OR 'coefficient':ab,ti OR 'internal consistency':ab,ti OR (cronbach*:ab,ti AND ('alpha':ab,ti OR 'alphas':ab,ti)) OR 'item correlation':ab,ti OR 'item correlations':ab,ti OR 'item selection':ab,ti OR 'item selections':ab,ti OR 'item reduction':ab,ti OR 'item reductions':ab,ti OR 'agreement':ab,ti OR 'precision':ab,ti OR 'imprecision':ab,ti OR 'precise values':ab,ti OR 'test-retest':ab,ti OR ('test':ab,ti AND 'retest':ab,ti) OR (reliab*:ab,ti AND ('test':ab,ti OR 'retest':ab,ti)) OR 'stability':ab,ti OR 'interrater':ab,ti OR 'inter-rater':ab,ti OR 'intrarater':ab,ti OR 'intra-rater':ab,ti OR 'intertester':ab,ti OR 'inter-tester':ab,ti OR 'intratester':ab,ti OR 'interobeserver':ab,ti OR 'inter-observer':ab,ti OR 'intraobserver':ab,ti OR 'intertechnician':ab,ti OR 'inter-technician':ab,ti OR 'intratechnician':ab,ti OR 'interexaminer':ab,ti OR 'inter-examiner':ab,ti OR 'intraexaminer':ab,ti OR 'interassay':ab,ti OR 'inter-assay':ab,ti OR 'intraassay':ab,ti OR 'intra-assay':ab,ti OR 'interindividual':ab,ti OR 'inter-individual':ab,ti OR 'intraindividual':ab,ti OR 'intra-individual':ab,ti OR 'interparticipant':ab,ti OR 'inter-participant':ab,ti OR 'intraparticipant':ab,ti OR 'kappa':ab,ti OR 'kappas':ab,ti OR 'coefficient of variation':ab,ti OR repeatab*:ab,ti OR ((replicab*:ab,ti OR 'repeated':ab,ti) AND ('measure':ab,ti OR 'measures':ab,ti OR 'findings':ab,ti OR 'result':ab,ti OR 'results':ab,ti OR 'test':ab,ti OR 'tests':ab,ti)) OR generaliza*:ab,ti OR generalisa*:ab,ti OR 'concordance':ab,ti OR ('intraclass':ab,ti AND correlation*:ab,ti) OR 'discriminative':ab,ti OR 'known group':ab,ti OR 'factor analysis':ab,ti OR 'factor analyses':ab,ti OR 'factor structure':ab,ti OR 'factor structures':ab,ti OR 'dimensionality':ab,ti OR subscale*:ab,ti OR 'multitrait scaling analysis':ab,ti OR 'multitrait scaling analyses':ab,ti OR 'item discriminant':ab,ti OR 'interscale correlation':ab,ti OR 'interscale correlations':ab,ti OR (('error':ab,ti OR 'errors':ab,ti) AND (measure*:ab,ti OR correlat*:ab,ti OR evaluat*:ab,ti OR 'accuracy':ab,ti OR 'accurate':ab,ti OR 'precision':ab,ti OR 'mean':ab,ti)) OR 'individual variability':ab,ti OR 'interval variability':ab,ti OR 'rate variability':ab,ti OR 'variability analysis':ab,ti OR ('uncertainty':ab,ti AND ('measurement':ab,ti OR 'measuring':ab,ti)) OR 'standard error of measurement':ab,ti OR sensitiv*:ab,ti OR responsive*:ab,ti OR ('limit':ab,ti AND 'detection':ab,ti) OR 'minimal detectable concentration':ab,ti OR interpretab*:ab,ti OR (small*:ab,ti AND ('real':ab,ti OR 'detectable':ab,ti) AND ('change':ab,ti OR 'difference':ab,ti)) OR 'meaningful change':ab,ti OR 'minimal important change':ab,ti OR 'minimal important difference':ab,ti OR 'minimally important change':ab,ti OR 'minimally important difference':ab,ti OR 'minimal detectable change':ab,ti OR 'minimal detectable difference':ab,ti OR 'minimally detectable change':ab,ti OR 'minimally detectable difference':ab,ti OR 'minimal real change':ab,ti OR 'minimal real difference':ab,ti OR 'minimally real change':ab,ti OR 'minimally real difference':ab,ti OR 'ceiling effect':ab,ti OR 'floor effect':ab,ti OR 'item response model':ab,ti OR 'irt':ab,ti OR 'rasch':ab,ti OR 'differential item functioning':ab,ti OR 'dif':ab,ti OR 'computer adaptive testing':ab,ti OR 'item bank':ab,ti OR 'cross-cultural equivalence':ab,ti) AND (handheld OR dynamometer OR isokinetic OR 'hand heald' OR 'hand-held') AND (flexion OR extension OR abduction OR (internal AND rotation) OR (rotation AND external) OR rotation OR adduction OR pronation OR supination OR eversion OR inversion OR (plantar AND flexion) OR dorsiflexion) AND (((strength OR muscular) AND strength OR musculoskeletal) AND strength OR isometric OR contraction OR muscle) AND (talocrural OR 'ankle joint' OR foot OR ankle)  #2 #1 AND [embase]/lim NOT ([embase]/lim AND [medline]/lim)  #3 #1 AND #2 |
| CINAHL | (((MH “Psychometrics”) or ( TI psychometr* or AB psychometr* ) or ( TI clinimetr* or AB clinimetr* ) or ( TI clinometr* OR AB clinometr* ) or (MH “Outcome Assessment”) or ( TI outcome assessment or AB outcome assessment ) or ( TI outcome measure* or AB outcome measure* ) or (MH “Health Status Indicators”) or (MH “Reproducibility of Results”) or (MH “Discriminant Analysis”) or ( ( TI reproducib* or AB reproducib* ) or ( TI reliab* or AB reliab* ) or ( TI unreliab* or AB unreliab* ) ) or ( ( TI valid* or AB valid* ) or ( TI coefficient or AB coefficient ) or ( TI homogeneity or AB homogeneity ) ) or ( TI homogeneous or AB homogeneous ) or ( TI “coefficient of variation” or AB “coefficient of variation” ) or ( TI “internal consistency” or AB “internal consistency” ) or (MH “Internal Consistency+”) or (MH “Reliability+”) or (MH “Measurement Error+”) or (MH “Content Validity+”) or “hypothesis testing” or “structural validity” or “cross-cultural validity” or (MH “Criterion-Related Validity+”) or “responsiveness” or “interpretability” or ( TI reliab* or AB reliab* ) and ( (TI test or AB test) OR (TI retest or AB retest) ) or ( TI stability or AB stability ) or ( TI interrater or AB interrater ) or ( TI inter-rater or AB inter-rater ) or ( TI intrarater or AB intrarater ) or ( TI intra-rater or AB intrarater ) or ( TI intertester or AB intertester) or (TI inter-tester or AB inter-tester) or ( TI intratester or AB intratester) or ( TI intra-tester or AB intra-tester) or ( TI interobserver or AB interobserver) or (TI inter-observer or AB inter-observer ) or ( TI intraobserver or AB intraobserver) or ( TI intra-observer or AB intra-observer) or ( TI intertechnician or AB intertechnician) or (TI inter-technician or AB inter-technician) or ( TI intratechnician or AB intratechnician ) or ( TI intra-technician or AB intra-technician ) or ( TI interexaminer or AB interexaminer ) or (TI inter-examiner or AB inter-examiner) or (TI intraexaminer or AB intraexaminer ) OR (TI intra-examiner or AB intra-examiner ) or (TI intra-examiner or AB intraexaminer ) or (TI interassay or AB interassay ) or ( TI inter-assay or AB inter-assay ) or ( TI intraassay or AB intraassay) or ( TI intra-assay or AB intra-assay ) or (TI interindividual or AB interindividual) or (TI inter-individual or AB inter-individual) OR (TI intraindividual or AB intraindividual) or (TI intra-individual or AB intra-individual) or (TI interparticipant or AB interparticipant) or (TI inter-participant or AB inter-participant ) or (TI intraparticipant or AB intraparticipant) or (TI intra-participant or AB intra-participant ) or (TI kappa or AB kappa) or (TI kappa’s or AB kappa’s ) or (TI kappas or AB kappas) or (TI repeatab* or AB repeatab*) or ( TI responsive* or AB responsive* ) or ( TI interpretab* or AB interpretab* )) AND (ankle or talocrural or ankle joint or foot) AND (handheld OR dynamometer OR Isokinetic OR hand-heald OR hand held)) AND (Flexion or extension or abduction or internal rotation or external rotation or adduction or pronation or supination or eversion or inversion or OR plantar flexion OR dorsiflexion) AND (strength or muscular strength or musculoskeletal strength OR isometric OR contraction or muscle) |
| Web of Science | Intrassession OR intra-examiner OR intra-rater OR inter-tester OR inter-rater OR Intra session OR reliability OR “intra tester” OR “intra examiner” OR “intra rater” OR “inter observer” OR “inter tester” OR “inter examiner” OR “inter rater” OR ICC OR Test-retest OR Validation OR “Reproducibility of Results” OR intra-session OR intra-tester OR intra-examiner OR intra-rater OR inter-observer OR inter-examiner OR icc intraclass OR intra class OR intra-class OR coefficient OR intraclass OR intra class OR intra-class OR correlation OR test-retest OR reliability OR validity OR reliability OR reproducibility  AND  talocrural OR 'ankle joint' OR foot OR ankle  AND  handheld OR dynamometer OR isokinetic OR 'hand held' OR 'hand-held'  AND  flexion OR extension OR abduction OR rotation OR adduction OR pronation OR supination OR eversion OR inversion OR “plantar flexion” OR dorsiflexion  AND  strength OR muscle OR musculoskeletal OR isometric OR contraction |
| SPORTDiscus | (instrumentation OR Validation Studies OR Comparative Study OR psychometrics OR psychometr* OR clinimetr* OR clinometr* OR outcome assessment health care OR outcome assessment OR outcome measure* OR observer variation OR observer variation OR Health Status Indicators OR reproducibility of results OR reproducib* OR discriminant analysis OR reliab* OR unreliab* OR valid* OR coefficient of variation OR coefficient OR homogeneity OR homogeneous OR internal consistency OR cronbach* OR test-retest OR test retest OR reliab* OR stability OR interrater OR inter-rater OR intrarater OR intra-rater OR intertester OR inter-tester OR intratester OR intra-tester OR interobserver OR inter-observer OR intraobserver OR intra-observer OR intertechnician OR inter-technician OR intratechnician OR intra-technician OR interexaminer OR inter-examiner OR OR factor structure OR factor structures OR dimension* OR subscale* OR item discriminant OR interscale correlation* OR individual variability OR interval variability OR rate variability OR measurement OR measuring OR standard error of measurement OR sensitiv* OR responsive* OR minimal detectable concentration OR interpretab* OR (minimal OR minimally OR clinical OR clinically) AND (important OR significant OR detectable) OR (small* detectable) AND (change OR difference))) AND (talocrural OR "ankle joint" OR foot OR ankle) AND (handheld OR dynamometer OR isokinetic OR 'hand held' OR 'hand-held') AND (flexion OR extension OR abduction OR rotation OR adduction OR pronation OR supination OR eversion OR inversion OR “plantar flexion” OR dorsiflexion) AND (strength OR muscle OR musculoskeletal OR isometric OR contraction) |
| PsycInfo | (instrumentation OR Validation Studies OR Comparative Study OR psychometrics OR psychometr* OR clinimetr* OR clinometr* OR outcome assessment health care OR outcome assessment OR outcome measure* OR observer variation OR observer variation OR Health Status Indicators OR reproducibility of results OR reproducib* OR discriminant analysis OR reliab* OR unreliab* OR valid* OR coefficient of variation OR coefficient OR homogeneity OR homogeneous OR internal consistency OR cronbach* OR test-retest OR test retest OR reliab* OR stability OR interrater OR inter-rater OR intrarater OR intra-rater OR intertester OR inter-tester OR intratester OR intra-tester OR interobserver OR inter-observer OR intraobserver OR intra-observer OR intertechnician OR inter-technician OR intratechnician OR intra-technician OR interexaminer OR inter-examiner OR OR factor structure OR factor structures OR dimension* OR subscale* OR item discriminant OR interscale correlation* OR individual variability OR interval variability OR rate variability OR measurement OR measuring OR standard error of measurement OR sensitiv* OR responsive* OR minimal detectable concentration OR interpretab* OR (minimal OR minimally OR clinical OR clinically) AND (important OR significant OR detectable) OR (small* detectable) AND (change OR difference))) AND (talocrural OR "ankle joint" OR foot OR ankle) AND (handheld OR dynamometer OR isokinetic OR 'hand held' OR 'hand-held') AND (flexion OR extension OR abduction OR rotation OR adduction OR pronation OR supination OR eversion OR inversion OR “plantar flexion” OR dorsiflexion) AND (strength OR muscle OR musculoskeletal OR isometric OR contraction) |
| SCOPUS | intrassession OR reliability OR intra-tester OR intra-examiner OR intra-rater OR inter-observer OR inter-tester OR inter-examiner OR inter-rater OR intra AND session OR reliability OR intra AND tester OR intra AND examiner OR intra AND rater OR inter AND observer OR inter AND tester OR inter AND examiner OR inter AND rater OR icc OR intraclass OR intra AND class OR "intra-class" OR test-retest OR validity OR reliability OR reliability OR validation OR "Reproducibility of Results" OR intra-session OR reliability OR intra-tester OR intra-examiner OR intra-rater OR inter-observer OR inter-tester OR inter-examiner OR icc AND intraclass OR intra AND class OR intra-class OR coefficient OR intraclass OR intra AND class OR intra-class OR correlation OR test-retest OR reliability OR validity OR reliability OR validity OR "validation study" OR reproducibility  AND  talocrural OR "ankle joint" OR foot OR ankle  AND  handheld OR dynamometer OR isokinetic OR 'hand AND held' OR 'hand-held'  AND  flexion OR extension OR abduction OR rotation OR adduction OR pronation OR supination OR eversion OR inversion OR "plantar flexion" OR dorsiflexion  AND  strength OR muscle OR musculoskeletal OR isometric OR contraction |
| BVS | ((Intrassession OR intra-examiner OR intra-rater OR inter-tester OR inter-rater OR Intra session OR reliability OR "intra tester" OR "intra examiner" OR "intra rater" OR "inter observer" OR "inter tester" OR "inter examiner" OR "inter rater" OR ICC OR Test-retest OR Validation OR "Reproducibility of Results" OR intra-session OR intra-tester OR intra-examiner OR intra-rater OR inter-observer OR inter-examiner OR icc intraclass OR intra class OR intra-class OR coefficient OR intraclass OR intra class OR intra-class OR correlation OR test-retest OR reliability OR validity OR reliability OR reproducibility) AND (talocrural OR "ankle joint" OR foot OR ankle) AND (handheld OR dynamometer OR isokinetic OR 'hand held' OR 'hand-held')) |

**Supplementary Table S2.** Updated search strategy (August 6, 2026)

| PubMed | (((((instrumentation[sh] OR methods[sh] OR "Validation Studies"[pt] OR "Comparative Study"[pt] OR "psychometrics"[MeSH] OR psychometr*[tiab] OR clinimetr*[tw] OR clinometr*[tw] OR "outcome assessment (health care)"[MeSH] OR "outcome assessment"[tiab] OR "outcome measure*"[tw] OR "observer variation"[MeSH] OR "observer variation"[tiab] OR "Health Status Indicators"[Mesh] OR "reproducibility of results"[MeSH] OR reproducib*[tiab] OR "discriminant analysis"[MeSH] OR reliab*[tiab] OR unreliab*[tiab] OR valid*[tiab] OR "coefficient of variation"[tiab] OR coefficient[tiab] OR homogeneity[tiab] OR homogeneous[tiab] OR "internal consistency"[tiab] OR (cronbach*[tiab] AND (alpha[tiab] OR alphas[tiab])) OR (item[tiab] AND (correlation*[tiab] OR selection*[tiab] OR reduction*[tiab])) OR agreement[tw] OR precision[tw] OR imprecision[tw] OR "precise values"[tw] OR test-retest[tiab] OR (test[tiab] AND retest[tiab]) OR (reliab*[tiab] AND (test[tiab] OR retest[tiab])) OR stability[tiab] OR interrater[tiab] OR inter-rater[tiab] OR intrarater[tiab] OR intra-rater[tiab] OR intertester[tiab] OR inter-tester[tiab] OR intratester[tiab] OR intra-tester[tiab] OR interobserver[tiab] OR inter-observer[tiab] OR intraobserver[tiab] OR intra-observer[tiab] OR intertechnician[tiab] OR inter-technician[tiab] OR intratechnician[tiab] OR intra-technician[tiab] OR interexaminer[tiab] OR inter-examiner[tiab] OR intraexaminer[tiab] OR intra-examiner[tiab] OR interassay[tiab] OR inter-assay[tiab] OR intraassay[tiab] OR intra-assay[tiab] OR interindividual[tiab] OR inter-individual[tiab] OR intraindividual[tiab] OR intra-individual[tiab] OR interparticipant[tiab] OR inter-participant[tiab] OR intraparticipant[tiab] OR intra-participant[tiab] OR kappa[tiab] OR kappa’s[tiab] OR kappas[tiab] OR repeatab*[tw] OR ((replicab*[tw] OR repeated[tw]) AND (measure[tw] OR measures[tw] OR findings[tw] OR result[tw] OR results[tw] OR test[tw] OR tests[tw])) OR generaliza*[tiab] OR generalisa*[tiab] OR concordance[tiab] OR (intraclass[tiab] AND correlation*[tiab]) OR discriminative[tiab] OR "known group"[tiab] OR "factor analysis"[tiab] OR "factor analyses"[tiab] OR "factor structure"[tiab] OR "factor structures"[tiab] OR dimension*[tiab] OR subscale*[tiab] OR (multitrait[tiab] AND scaling[tiab] AND (analysis[tiab] OR analyses[tiab])) OR "item discriminant"[tiab] OR "interscale correlation*"[tiab] OR error[tiab] OR errors[tiab] OR "individual variability"[tiab] OR "interval variability"[tiab] OR "rate variability"[tiab] OR (variability[tiab] AND (analysis[tiab] OR values[tiab])) OR (uncertainty[tiab] AND (measurement[tiab] OR measuring[tiab])) OR "standard error of measurement"[tiab] OR sensitiv*[tiab] OR responsive*[tiab] OR (limit[tiab] AND detection[tiab]) OR "minimal detectable concentration"[tiab] OR interpretab*[tiab] OR ((minimal[tiab] OR minimally[tiab] OR clinical[tiab] OR clinically[tiab]) AND (important[tiab] OR significant[tiab] OR detectable[tiab]) AND (change[tiab] OR difference[tiab])) OR (small*[tiab] AND (real[tiab] OR detectable[tiab]) AND (change[tiab] OR difference[tiab])) OR "meaningful change"[tiab] OR "ceiling effect"[tiab] OR "floor effect"[tiab] OR "Item response model"[tiab] OR IRT[tiab] OR Rasch[tiab] OR "Differential item functioning"[tiab] OR DIF[tiab] OR "computer adaptive testing"[tiab] OR "item bank"[tiab] OR "cross-cultural equivalence"[tiab])) AND (talocrural OR "ankle joint" OR foot OR ankle)) AND (handheld OR dynamometer OR isokinetic OR 'hand held' OR 'hand-held')) AND (flexion OR extension OR abduction OR (internal AND rotation) OR (rotation AND external) OR rotation OR adduction OR pronation OR supination OR eversion OR inversion OR (plantar flexion) OR dorsiflexion)) AND (strength OR muscle OR musculoskeletal OR isometric OR contraction)  Filters applied: from 2024/12/9 - 2026/8/6. |
| --- | --- |
| EMBASE | #1 ('intermethod comparison'/exp OR 'data collection method'/exp OR 'validation study'/exp OR 'feasibility study'/exp OR 'pilot study'/exp OR 'psychometry'/exp OR 'reproducibility'/exp OR reproducib*:ab,ti OR 'audit':ab,ti OR psychometr*:ab,ti OR clinimetr*:ab,ti OR clinometr*:ab,ti OR 'observer variation'/exp OR 'observer variation':ab,ti OR 'discriminant analysis'/exp OR 'validity'/exp OR reliab*:ab,ti OR valid*:ab,ti OR 'coefficient':ab,ti OR 'internal consistency':ab,ti OR (cronbach*:ab,ti AND ('alpha':ab,ti OR 'alphas':ab,ti)) OR 'item correlation':ab,ti OR 'item correlations':ab,ti OR 'item selection':ab,ti OR 'item selections':ab,ti OR 'item reduction':ab,ti OR 'item reductions':ab,ti OR 'agreement':ab,ti OR 'precision':ab,ti OR 'imprecision':ab,ti OR 'precise values':ab,ti OR 'test-retest':ab,ti OR ('test':ab,ti AND 'retest':ab,ti) OR (reliab*:ab,ti AND ('test':ab,ti OR 'retest':ab,ti)) OR 'stability':ab,ti OR 'interrater':ab,ti OR 'inter-rater':ab,ti OR 'intrarater':ab,ti OR 'intra-rater':ab,ti OR 'intertester':ab,ti OR 'inter-tester':ab,ti OR 'intratester':ab,ti OR 'interobeserver':ab,ti OR 'inter-observer':ab,ti OR 'intraobserver':ab,ti OR 'intertechnician':ab,ti OR 'inter-technician':ab,ti OR 'intratechnician':ab,ti OR 'interexaminer':ab,ti OR 'inter-examiner':ab,ti OR 'intraexaminer':ab,ti OR 'interassay':ab,ti OR 'inter-assay':ab,ti OR 'intraassay':ab,ti OR 'intra-assay':ab,ti OR 'interindividual':ab,ti OR 'inter-individual':ab,ti OR 'intraindividual':ab,ti OR 'intra-individual':ab,ti OR 'interparticipant':ab,ti OR 'inter-participant':ab,ti OR 'intraparticipant':ab,ti OR 'kappa':ab,ti OR 'kappas':ab,ti OR 'coefficient of variation':ab,ti OR repeatab*:ab,ti OR ((replicab*:ab,ti OR 'repeated':ab,ti) AND ('measure':ab,ti OR 'measures':ab,ti OR 'findings':ab,ti OR 'result':ab,ti OR 'results':ab,ti OR 'test':ab,ti OR 'tests':ab,ti)) OR generaliza*:ab,ti OR generalisa*:ab,ti OR 'concordance':ab,ti OR ('intraclass':ab,ti AND correlation*:ab,ti) OR 'discriminative':ab,ti OR 'known group':ab,ti OR 'factor analysis':ab,ti OR 'factor analyses':ab,ti OR 'factor structure':ab,ti OR 'factor structures':ab,ti OR 'dimensionality':ab,ti OR subscale*:ab,ti OR 'multitrait scaling analysis':ab,ti OR 'multitrait scaling analyses':ab,ti OR 'item discriminant':ab,ti OR 'interscale correlation':ab,ti OR 'interscale correlations':ab,ti OR (('error':ab,ti OR 'errors':ab,ti) AND (measure*:ab,ti OR correlat*:ab,ti OR evaluat*:ab,ti OR 'accuracy':ab,ti OR 'accurate':ab,ti OR 'precision':ab,ti OR 'mean':ab,ti)) OR 'individual variability':ab,ti OR 'interval variability':ab,ti OR 'rate variability':ab,ti OR 'variability analysis':ab,ti OR ('uncertainty':ab,ti AND ('measurement':ab,ti OR 'measuring':ab,ti)) OR 'standard error of measurement':ab,ti OR sensitiv*:ab,ti OR responsive*:ab,ti OR ('limit':ab,ti AND 'detection':ab,ti) OR 'minimal detectable concentration':ab,ti OR interpretab*:ab,ti OR (small*:ab,ti AND ('real':ab,ti OR 'detectable':ab,ti) AND ('change':ab,ti OR 'difference':ab,ti)) OR 'meaningful change':ab,ti OR 'minimal important change':ab,ti OR 'minimal important difference':ab,ti OR 'minimally important change':ab,ti OR 'minimally important difference':ab,ti OR 'minimal detectable change':ab,ti OR 'minimal detectable difference':ab,ti OR 'minimally detectable change':ab,ti OR 'minimally detectable difference':ab,ti OR 'minimal real change':ab,ti OR 'minimal real difference':ab,ti OR 'minimally real change':ab,ti OR 'minimally real difference':ab,ti OR 'ceiling effect':ab,ti OR 'floor effect':ab,ti OR 'item response model':ab,ti OR 'irt':ab,ti OR 'rasch':ab,ti OR 'differential item functioning':ab,ti OR 'dif':ab,ti OR 'computer adaptive testing':ab,ti OR 'item bank':ab,ti OR 'cross-cultural equivalence':ab,ti) AND (handheld OR dynamometer OR isokinetic OR 'hand heald' OR 'hand-held') AND (flexion OR extension OR abduction OR (internal AND rotation) OR (rotation AND external) OR rotation OR adduction OR pronation OR supination OR eversion OR inversion OR (plantar AND flexion) OR dorsiflexion) AND (((strength OR muscular) AND strength OR musculoskeletal) AND strength OR isometric OR contraction OR muscle) AND (talocrural OR 'ankle joint' OR foot OR ankle)  #2 #1 AND [embase]/lim NOT ([embase]/lim AND [medline]/lim)  #3 #1 AND #2  # #3 AND (2024:py OR 2025:py OR 2026:py) |
| CINAHL | (((MH “Psychometrics”) or ( TI psychometr* or AB psychometr* ) or ( TI clinimetr* or AB clinimetr* ) or ( TI clinometr* OR AB clinometr* ) or (MH “Outcome Assessment”) or ( TI outcome assessment or AB outcome assessment ) or ( TI outcome measure* or AB outcome measure* ) or (MH “Health Status Indicators”) or (MH “Reproducibility of Results”) or (MH “Discriminant Analysis”) or ( ( TI reproducib* or AB reproducib* ) or ( TI reliab* or AB reliab* ) or ( TI unreliab* or AB unreliab* ) ) or ( ( TI valid* or AB valid* ) or ( TI coefficient or AB coefficient ) or ( TI homogeneity or AB homogeneity ) ) or ( TI homogeneous or AB homogeneous ) or ( TI “coefficient of variation” or AB “coefficient of variation” ) or ( TI “internal consistency” or AB “internal consistency” ) or (MH “Internal Consistency+”) or (MH “Reliability+”) or (MH “Measurement Error+”) or (MH “Content Validity+”) or “hypothesis testing” or “structural validity” or “cross-cultural validity” or (MH “Criterion-Related Validity+”) or “responsiveness” or “interpretability” or ( TI reliab* or AB reliab* ) and ( (TI test or AB test) OR (TI retest or AB retest) ) or ( TI stability or AB stability ) or ( TI interrater or AB interrater ) or ( TI inter-rater or AB inter-rater ) or ( TI intrarater or AB intrarater ) or ( TI intra-rater or AB intrarater ) or ( TI intertester or AB intertester) or (TI inter-tester or AB inter-tester) or ( TI intratester or AB intratester) or ( TI intra-tester or AB intra-tester) or ( TI interobserver or AB interobserver) or (TI inter-observer or AB inter-observer ) or ( TI intraobserver or AB intraobserver) or ( TI intra-observer or AB intra-observer) or ( TI intertechnician or AB intertechnician) or (TI inter-technician or AB inter-technician) or ( TI intratechnician or AB intratechnician ) or ( TI intra-technician or AB intra-technician ) or ( TI interexaminer or AB interexaminer ) or (TI inter-examiner or AB inter-examiner) or (TI intraexaminer or AB intraexaminer ) OR (TI intra-examiner or AB intra-examiner ) or (TI intra-examiner or AB intraexaminer ) or (TI interassay or AB interassay ) or ( TI inter-assay or AB inter-assay ) or ( TI intraassay or AB intraassay) or ( TI intra-assay or AB intra-assay ) or (TI interindividual or AB interindividual) or (TI inter-individual or AB inter-individual) OR (TI intraindividual or AB intraindividual) or (TI intra-individual or AB intra-individual) or (TI interparticipant or AB interparticipant) or (TI inter-participant or AB inter-participant ) or (TI intraparticipant or AB intraparticipant) or (TI intra-participant or AB intra-participant ) or (TI kappa or AB kappa) or (TI kappa’s or AB kappa’s ) or (TI kappas or AB kappas) or (TI repeatab* or AB repeatab*) or ( TI responsive* or AB responsive* ) or ( TI interpretab* or AB interpretab* )) AND (ankle or talocrural or ankle joint or foot) AND (handheld OR dynamometer OR Isokinetic OR hand-heald OR hand held)) AND (Flexion or extension or abduction or internal rotation or external rotation or adduction or pronation or supination or eversion or inversion or OR plantar flexion OR dorsiflexion) AND (strength or muscular strength or musculoskeletal strength OR isometric OR contraction or muscle)  12/2024 – 08/2026 |
| Web of Science | Intrassession OR intra-examiner OR intra-rater OR inter-tester OR inter-rater OR Intra session OR reliability OR “intra tester” OR “intra examiner” OR “intra rater” OR “inter observer” OR “inter tester” OR “inter examiner” OR “inter rater” OR ICC OR Test-retest OR Validation OR “Reproducibility of Results” OR intra-session OR intra-tester OR intra-examiner OR intra-rater OR inter-observer OR inter-examiner OR icc intraclass OR intra class OR intra-class OR coefficient OR intraclass OR intra class OR intra-class OR correlation OR test-retest OR reliability OR validity OR reliability OR reproducibility  AND  talocrural OR 'ankle joint' OR foot OR ankle  AND  handheld OR dynamometer OR isokinetic OR 'hand held' OR 'hand-held'  AND  flexion OR extension OR abduction OR rotation OR adduction OR pronation OR supination OR eversion OR inversion OR “plantar flexion” OR dorsiflexion  AND  strength OR muscle OR musculoskeletal OR isometric OR contraction  2024-12-09 to 2026-08-06 |
| SPORTDiscus | (instrumentation OR Validation Studies OR Comparative Study OR psychometrics OR psychometr* OR clinimetr* OR clinometr* OR outcome assessment health care OR outcome assessment OR outcome measure* OR observer variation OR observer variation OR Health Status Indicators OR reproducibility of results OR reproducib* OR discriminant analysis OR reliab* OR unreliab* OR valid* OR coefficient of variation OR coefficient OR homogeneity OR homogeneous OR internal consistency OR cronbach* OR test-retest OR test retest OR reliab* OR stability OR interrater OR inter-rater OR intrarater OR intra-rater OR intertester OR inter-tester OR intratester OR intra-tester OR interobserver OR inter-observer OR intraobserver OR intra-observer OR intertechnician OR inter-technician OR intratechnician OR intra-technician OR interexaminer OR inter-examiner OR OR factor structure OR factor structures OR dimension* OR subscale* OR item discriminant OR interscale correlation* OR individual variability OR interval variability OR rate variability OR measurement OR measuring OR standard error of measurement OR sensitiv* OR responsive* OR minimal detectable concentration OR interpretab* OR (minimal OR minimally OR clinical OR clinically) AND (important OR significant OR detectable) OR (small* detectable) AND (change OR difference))) AND (talocrural OR "ankle joint" OR foot OR ankle) AND (handheld OR dynamometer OR isokinetic OR 'hand held' OR 'hand-held') AND (flexion OR extension OR abduction OR rotation OR adduction OR pronation OR supination OR eversion OR inversion OR “plantar flexion” OR dorsiflexion) AND (strength OR muscle OR musculoskeletal OR isometric OR contraction)  12/2024 – 08/2026 |
| PsycInfo | (instrumentation OR Validation Studies OR Comparative Study OR psychometrics OR psychometr* OR clinimetr* OR clinometr* OR outcome assessment health care OR outcome assessment OR outcome measure* OR observer variation OR observer variation OR Health Status Indicators OR reproducibility of results OR reproducib* OR discriminant analysis OR reliab* OR unreliab* OR valid* OR coefficient of variation OR coefficient OR homogeneity OR homogeneous OR internal consistency OR cronbach* OR test-retest OR test retest OR reliab* OR stability OR interrater OR inter-rater OR intrarater OR intra-rater OR intertester OR inter-tester OR intratester OR intra-tester OR interobserver OR inter-observer OR intraobserver OR intra-observer OR intertechnician OR inter-technician OR intratechnician OR intra-technician OR interexaminer OR inter-examiner OR OR factor structure OR factor structures OR dimension* OR subscale* OR item discriminant OR interscale correlation* OR individual variability OR interval variability OR rate variability OR measurement OR measuring OR standard error of measurement OR sensitiv* OR responsive* OR minimal detectable concentration OR interpretab* OR (minimal OR minimally OR clinical OR clinically) AND (important OR significant OR detectable) OR (small* detectable) AND (change OR difference))) AND (talocrural OR "ankle joint" OR foot OR ankle) AND (handheld OR dynamometer OR isokinetic OR 'hand held' OR 'hand-held') AND (flexion OR extension OR abduction OR rotation OR adduction OR pronation OR supination OR eversion OR inversion OR “plantar flexion” OR dorsiflexion) AND (strength OR muscle OR musculoskeletal OR isometric OR contraction)  2024-2026 |
| SCOPUS | intrassession OR reliability OR intra-tester OR intra-examiner OR intra-rater OR inter-observer OR inter-tester OR inter-examiner OR inter-rater OR intra AND session OR reliability OR intra AND tester OR intra AND examiner OR intra AND rater OR inter AND observer OR inter AND tester OR inter AND examiner OR inter AND rater OR icc OR intraclass OR intra AND class OR "intra-class" OR test-retest OR validity OR reliability OR reliability OR validation OR "Reproducibility of Results" OR intra-session OR reliability OR intra-tester OR intra-examiner OR intra-rater OR inter-observer OR inter-tester OR inter-examiner OR icc AND intraclass OR intra AND class OR intra-class OR coefficient OR intraclass OR intra AND class OR intra-class OR correlation OR test-retest OR reliability OR validity OR reliability OR validity OR "validation study" OR reproducibility  AND  talocrural OR "ankle joint" OR foot OR ankle  AND  handheld OR dynamometer OR isokinetic OR 'hand AND held' OR 'hand-held'  AND  flexion OR extension OR abduction OR rotation OR adduction OR pronation OR supination OR eversion OR inversion OR "plantar flexion" OR dorsiflexion  AND  strength OR muscle OR musculoskeletal OR isometric OR contraction  2024-2026 |
| BVS | ((Intrassession OR intra-examiner OR intra-rater OR inter-tester OR inter-rater OR Intra session OR reliability OR "intra tester" OR "intra examiner" OR "intra rater" OR "inter observer" OR "inter tester" OR "inter examiner" OR "inter rater" OR ICC OR Test-retest OR Validation OR "Reproducibility of Results" OR intra-session OR intra-tester OR intra-examiner OR intra-rater OR inter-observer OR inter-examiner OR icc intraclass OR intra class OR intra-class OR coefficient OR intraclass OR intra class OR intra-class OR correlation OR test-retest OR reliability OR validity OR reliability OR reproducibility) AND (talocrural OR "ankle joint" OR foot OR ankle) AND (handheld OR dynamometer OR isokinetic OR 'hand held' OR 'hand-held'))  2024-2026 |

**Supplementary Table S3.** Studies excluded after full-text assessment and reasons for exclusion.

| **Author (Year)** | **DOI / PMID** | **PRISMA Reason for Exclusion** |
| --- | --- | --- |
| Bergdal et al. (2026) | 10.1016/j.jbmt.2025.10.054 | Wrong measurement protocol (combined inversion + plantar flexion) |
| Wang et al. (2025) | 10.1038/s41598-025-11664-w PMID: 40681760 | Wrong intervention (device is not a handheld dynamometer) |
| Stouge et al. (2026) | 10.1002/nbm.70188 PMID: 41276274 | Wrong intervention (device is not a handheld dynamometer) |
| Coventry et al. (2026) | 10.3390/jfmk11010116 PMID: 41900523 | Wrong intervention (device is not a handheld dynamometer) |

**Supplementary Figure S1.** Forest plots summarizing the inter-rater reliability (ICC 2,1) of ankle invertor and evertor muscle strength, shown in Panel A and Panel B respectively.

**Supplementary Figure S2.** Forest plots summarizing the intra-rater reliability (ICC 3,1) of dorsiflexor muscle strength. Panel A presents the overall meta-analysis including all studies. Panel B shows the subgroup analysis according to the presence or absence of external fixation.

**Supplementary Figure S3.** **Forest plots summarizing the intra-rater reliability (ICC 3.1) of plantar flexor muscle strength.** Panel A presents the overall meta-analysis including all studies. Panel B shows the subgroup analysis according to the presence or absence of external fixation.

**Supplementary Figure S4.** Forest plots summarizing the intra-rater reliability (ICC 2,1) of ankle invertor and evertor muscle strength, shown in Panel A and Panel B respectively.

**Supplementary Table S4.** ICC reliability classification.

| **Type of Reliability** | **ICC Model** | **Movement** | **Pooled ICC** | **Pooled 95% CI** | **Classification** |
| --- | --- | --- | --- | --- | --- |
| Inter-tester | ICC (2,1) | Dorsiflexion | 0.89 | 0.80–0.94 | Good |
| Intra-tester | ICC (3,1) | Dorsiflexion | 0.88 | 0.77–0.94 | Good |
| Inter-tester | ICC (2,1) | Plantar flexion | 0.86 | 0.72–0.93 | Good |
| Intra-tester | ICC (3,1) | Plantar flexion | 0.91 | 0.84–0.95 | Excellent |
| Inter-tester | ICC (2,1) | Invertors | 0.79 | 0.66–0.87 | Moderate to good |
| Intra-tester | ICC (3,1) | Invertors | 0.88 | 0.72–0.95 | Good |
| Inter-tester | ICC (2,1) | Evertors | 0.75 | 0.46–0.90 | Moderate (wide CI) |
| Intra-tester | ICC (3,1) | Evertors | 0.88 | 0.83–0.91 | Good |

**Abbreviations:** ICC, intraclass correlation coefficient; CI, confidence interval.

**Note:** Classification follows Koo & Li (2016). When the lower bound of the 95% CI falls into a lower category, a range is reported (e.g., moderate to good).

**Supplementary Table S5.** Pooled concurrent validity of hand-held dynamometry for ankle strength assessment.

| **Type of validity** | **Comparison method** | **Movement** | **Pooled Pearson’s r** | **Pooled 95% CI** | **Classification** |
| --- | --- | --- | --- | --- | --- |
| Concurrent validity | Isokinetic dynamometer | Dorsiflexion | 0.87 | 0.42–0.98 | Strong |
| Concurrent validity | Isokinetic dynamometer | Plantar flexion | 0.72 | −0.20–0.96 | * |

**Abbreviation:** CI, confidence interval.

*Classification not assigned because the 95% CI crosses zero, indicating substantial uncertainty in the pooled estimate.

**Supplementary Table S6.** Detailed COSMIN Risk of Bias judgments and main reasons for rating

| **Study** | **Overall Risk of Bias** | **Main Reason(s) for Judgment/Downgrading** |
| --- | --- | --- |
| Katoh & Yamasaki, 2009 | Very Good | External fixation present; standardized protocol; adequate sample size |
| Spink et al., 2010 | Very Good | External fixation present; standardized protocol; adequate sample size |
| Mahaffey et al., 2022 | Very Good | Clear concurrent validity design; adequate sample size |
| McGirr et al., 2014 | Adequate | Adequate sample size; only intra-tester reliability assessed; no external fixation |
| Daloia et al., 2018 | Adequate | Population restricted to children and adolescents, resulting in indirectness |
| Ford-Smith et al., 2001 | Adequate | Incomplete reporting of methodological items |
| Jackson et al., 2017 | Adequate | Small sample size |
| Kimura et al., 2018 | Adequate | External fixation present; incomplete reporting of some methodological items |
| Mauracher et al., 2019 | Adequate | Small sample size |
| McMahon et al., 1992 | Adequate | Limited methodological reporting |
| Romero-Franco et al., 2017 | Adequate | Small sample size |
| Schaeffer et al., 2021 | Adequate | No external fixation |
| Shimizu et al., 2023 | Adequate | External fixation present; small sample size |
| Buckinx et al., 2015 | Adequate | Older adult population; incomplete reporting of methodological items |
| Espino et al., 2025 | Adequate | External fixation present (belt-stabilized); multiple raters; small sample size |
| Nakagawa et al., 2025 | Doubtful | External fixation present; heel float reported; low concurrent validity |
| Kelln et al., 2008 | Doubtful | Potential influence of tester strength; moderate sample size |
| Kim & Lee, 2015 | Doubtful | Incomplete methodological reporting |
| Mentiplay et al., 2015 | Doubtful | No external fixation; lower concurrent validity for ankle muscles |
| Clarke et al., 2011 | Doubtful | Very low inter-tester reliability for plantar flexors |
| Rawdon et al., 2025 | Doubtful | Small sample size |
| Hébert et al., 2011 | Inadequate | Youth population only; small reliability subsample |
| Sanada et al., 2008 | Inadequate | Very small sample size |
| Kimura et al., 1996 | Inadequate | Very small sample size; exclusive use of the break test |

**Supplementary Table S7.** Sufficiency judgements of the measurement properties according to COSMIN criteria.

| **Measurement property** | **Sufficient (+)** | **Insufficient (−)** | **Indeterminate (?)** | **Not assessed** | **Total studies** |
| --- | --- | --- | --- | --- | --- |
| Reliability (intra- or inter-rater) | 21 | 1 | 0 | 2 | 24 |
| Measurement error (SEM/MDC) | 10 | 0 | 2 | 12 | 24 |
| Construct validity (HHD vs IKD) | 0 | 1 | 4 | 19 | 24 |

**Notes:**

Construct validity was rated according to the a priori hypotheses defined in section 2.7 (mainly r ≥ 0.70 for dorsiflexors – H1). Only H1 could be formally evaluated. Hypotheses H2 (stronger correlations with external fixation), H3 (higher protocol similarity leading to higher correlations) and H4 (lower correlations for plantar flexion than dorsiflexion) could not be tested due to insufficient comparable data across studies. Because fewer than 75% of the pre-specified hypotheses were confirmed, construct validity was judged as indeterminate for most study–property pairs. Nakagawa et al. (2025) was rated insufficient for plantar flexors (r = 0.34).

**Supplementary Table S8.** Certainty of evidence for the measurement properties of hand-held dynamometry.

| **Measurement property** | **Evidence base** | **Summarized rating** | **Downgrade reason(s)** | **Certainty of evidence** |
| --- | --- | --- | --- | --- |
| Reliability | 22 studies; results predominantly sufficient | Sufficient (+) | No downgrade applied. Although statistical heterogeneity was high (I² 85–92%), the direction of effects was consistent across studies and the lower bounds of most pooled estimates remained ≥ 0.70. | ⨁⨁⨁⨁ High |
| Measurement error | Studies reporting SEM and/or MDC | Indeterminate (?) | MIC was not established; therefore, sufficiency of measurement error could not be determined | Not graded |
| Construct validity | 5 unique studies comparing HHD with IKD | Indeterminate (?), with substantial uncertainty | Very serious inconsistency across studies and muscle groups (I² ≈ 92%); fewer than 75% of a priori hypotheses could be formally tested | ⨁⨁◯◯ Low |

**Abbreviations:** HHD, hand-held dynamometer; IKD, isokinetic dynamometer; MDC, minimal detectable change; MIC, minimal important change; SEM, standard error of measurement.

**Notes:**

High certainty for reliability was retained despite substantial statistical heterogeneity because the direction of effects was consistent (all point estimates indicated at least moderate reliability), the lower limits of the confidence intervals for the main muscle groups (dorsiflexors and plantar flexors) remained within the “good” range, and no serious risk of bias or indirectness was judged to further reduce confidence in the body of evidence.
